# Mitochondrial fission augments capsaicin-induced axonal degeneration

**DOI:** 10.1007/s00401-014-1354-3

**Published:** 2014-10-17

**Authors:** Hao Chiang, Nobuhiko Ohno, Yu-Lin Hsieh, Don J. Mahad, Shin Kikuchi, Hitoshi Komuro, Sung-Tsang Hsieh, Bruce D. Trapp

**Affiliations:** 1 Department of Anatomy and Cell Biology, National Taiwan University College of Medicine, Taipei, 10051 Taiwan; 2 Department of Neurosciences, Lerner Research Institute, Cleveland Clinic, Cleveland, OH 44195 USA; 3 Department of Neurology, National Taiwan University Hospital, Taipei, 10002 Taiwan; 4 Present Address: Department of Anatomy and Molecular Histology, University of Yamanashi, Chuo, Yamanashi 409-3898 Japan; 5 Present Address: Department of Anatomy, School of Medicine, College of Medicine, Kaohsiung Medical University, Kaohsiung, 80708 Taiwan; 6 Present Address: Centre for Neuroregeneration, University of Edinburgh, Chancellor’s Building, Edinburgh, 49 Little France Crescent, Edinburgh, EH16 4SB UK; 7 Present Address: Department of Anatomy 1, Sapporo Medical University School of Medicine, West 17, South 1, Chuo-ku, Sapporo, 060-8556 Japan

**Keywords:** Mitochondria, Axonal swellings, Capsaicin, Miro, Drp1

## Abstract

Capsaicin, an agonist of transient receptor potential vanilloid receptor 1, induces axonal degeneration of peripheral sensory nerves and is commonly used to treat painful sensory neuropathies. In this study, we investigated the role of mitochondrial dynamics in capsaicin-induced axonal degeneration. In capsaicin-treated rodent sensory axons, axonal swellings, decreased mitochondrial stationary site length and reduced mitochondrial transport preceded axonal degeneration. Increased axoplasmic Ca^2+^ mediated the alterations in mitochondrial length and transport. While sustaining mitochondrial transport did not reduce axonal swellings in capsaicin-treated axons, preventing mitochondrial fission by overexpression of mutant dynamin-related protein 1 increased mitochondrial length, retained mitochondrial membrane potentials and reduced axonal loss upon capsaicin treatment. These results establish that mitochondrial stationary site size significantly affects axonal integrity and suggest that inhibition of Ca^2+^-dependent mitochondrial fission facilitates mitochondrial function and axonal survival following activation of axonal cationic channels.

**Electronic supplementary material:**

The online version of this article (doi:10.1007/s00401-014-1354-3) contains supplementary material, which is available to authorized users.

## Introduction

Capsaicin (8-methyl-*N*-vanillyl-6-noneamide), a chili pepper irritant [71], is commonly used as a local analgesic in individuals with sensory nerve disorders such as diabetic neuropathy [7, 18]. By the activation of the transient receptor potential vanilloid receptor 1 (TRPV1), which is expressed on sensory axons [9, 43], capsaicin induces a nonselective cationic influx, leading to Ca^2+^-dependent desensitization and eventually local denervation of the skin [3, 23, 45, 47]. Previous studies have described the effects of capsaicin on neuronal cell bodies [16, 36], but less is known about the cellular and molecular alterations that cause distal axonal degeneration.

Sensory axons that innervate the skin originate from dorsal root ganglia (DRG) neurons and can attain a length which can exceed 1 m in adult humans. When acutely challenged by environmental or physiological stress, the integrity of distal axons must be acutely independent of neuronal gene transcription [56]. One critical component of axonal survival is the generation of ATP, which is essential for axonal metabolism, ion homeostasis, and nerve conduction [32, 73]. In addition, Na^+^/Ca^2+^ overloads that often follow energy deficits exert a negative impact on axonal survival [67]. An early pathologic hallmark of axonal degeneration is the formation of axonal swellings [67, 77]. Focal accumulation of mitochondria and cytoskeleton in axonal swellings suggests that energy failure and Ca^2+^ overload play pivotal roles in axonal degeneration [5, 42, 50].

Mitochondria, the major source of axonal ATP, respond to changes in axonal energy demand by altering their size, distribution, and motility [13, 61]. The majority of axonal mitochondria are stationary and enriched at sites of high ATP demand [37, 52]. By fusing with stationary mitochondria, a less abundant motile mitochondrial pool regulates stationary mitochondrial turnover and distribution [37, 62]. Motile mitochondria can also bud from stationary mitochondria by fission [2]. Dynamin-related protein 1 (Drp1), a soluble axoplasmic protein, regulates mitochondrial fission in an energy-dependent manner and a point mutation in its GTPase domain abolishes fission upon an increase in axoplasmic Ca^2+^ [12, 26, 41, 66]. While axonal degeneration and impaired mitochondrial fusion are features of patients with mutations in the mitochondrial proteins OPA1 and Mitofusin 2 [1, 81], it remains to be determined if changes in mitochondrial dynamics and/or rates of transport are involved in capsaicin-induced axonal degeneration.

To explore whether mitochondrial size and/or motility play a role in capsaicin-induced axonal degeneration, we examined sensory axonal pathology following capsaicin injection into mouse hindpaws in vivo and investigated how axons and axonal mitochondria respond after capsaicin treatment of DRG axons in vitro. Capsaicin activation of axonal TRPV1 caused axonal ovoid formation and subsequent axonal degeneration in vivo and in vitro. Reduced mitochondrial length in axonal swellings, which was dependent on Ca^2+^, suggests a key role of mitochondrial fission in capsaicin-induced axonal swellings and degeneration. In capsaicin-treated axons, inhibition of axonal mitochondrial fission by mutant Drp1 (Drp1K38A) sustains the length of mitochondrial stationary sites, increased mitochondrial membrane potentials, and significantly reduced axonal swellings and axonal degeneration. These data support the concept that inhibition of mitochondrial fission increases axonal mitochondrial size and reduces capsaicin-induced axonal degeneration.

## Materials and methods

### Intradermal injections of capsaicin

One hindpaw of 8-week-old male ICR mice (purchased from National Taiwan University College of Medicine Animal Center) received 2–7 daily intradermal injections of 1 % (w/v) capsaicin (CAP, Sigma, St. Louis, MO) dissolved in 10 % ethanol, 10 % Tween-80 and saline [27]. Vehicle solution was injected into the other hindpaw and served as a control. Minor swelling occurred at the injection site but resolved several hours later. All animal procedures were approved by the animal committee of National Taiwan University College of Medicine and were conducted according to Guide for the Care and Use of Laboratory Animals from National Research Council.

### Embryonic primary DRG culture and drug treatment

Dorsal root ganglia (DRG) cultures were produced from E16 to E17 Sprague–Dawley rats and maintained as described previously with minor modifications [37]. (See Online Resource 1: Supplemental Methods for additional details).

DRG cultures were incubated with either capsaicin (Sigma) or control solution (DMSO, Sigma) for 15 min, replenished with fresh medium, and further incubated for 1.5 or 48 h to evaluate axonal swellings and axonal loss, respectively. Serial capsaicin concentrations of 1, 10, 100 μM and 1 mM were tested to optimize treatment conditions and 100 μM capsaicin was used in subsequent experiments because it induced axonal ovoid formation and alterations in axonal mitochondrial behavior without causing axonal loss during the 15-min incubation period. In a set of experiments, 10 μM capsazepine (CZP, Sigma), a capsaicin antagonist, was added to DRG cultures for 10 min before the addition of capsaicin or DMSO.

### Lentiviral transfection

pLenti6/V5 vectors (Invitrogen) and pLVX vectors (Clontech) were used to transfect DRG neurons in vitro. These vectors included constructs to label mitochondria (Mito-DsRed and Mito-Dendra2), GCaMP3 for detecting axoplasmic Ca^2+^, wild-type (WT) and mutated mitochondrial Rho GTPase (Miro) to manipulate mitochondrial motility, and WT and mutated Drp1 to manipulate mitochondrial fission. Details of these constructs can be found in Online Resource 1: Supplemental Methods.

### Time-lapse imaging of mitochondrial dynamics, axoplasmic Ca^2+^, and mitochondrial membrane potentials

Time-lapse imaging of mitochondrial dynamics was performed using an inverted confocal microscope as described previously [37]. Stationary and motile mitochondria were identified and studied with Kymographs generated from axons transfected with Mito-DsRed or Mito-Dendra2. Increases in axoplasmic Ca^2+^ were measured by the fluorescence intensity of GCaMP3 as described previously [52] and the membrane potential of axonal mitochondria was determined in DRG cultures stained with tetramethylrhodamine methyl ester (TMRM, Invitrogen) as previously described with some modifications [75]. Details of these methods and other experimental conditions are described in Online Resource 1: Supplemental Methods.

### Axonal pathology

Hindpaw skin and associated medial plantar nerves from the injection sites were resected after transcardial perfusion with 4 % paraformaldehyde for light microscopy and 5 % glutaraldehyde for electron microscopy. DRG cultures were similarly immersion fixed. Epidermal and dermal nerves in the hindpaw skin and DRG cultures were immunochemically stained for protein gene product 9.5 (PGP9.5) [31, 70] and β-tubulin isotype III antibodies as markers for axonal integrity. Axonal swellings were defined as axonal spheroids which had single or dual axonal connections and were at least twice as wide as adjacent areas of the same axons. Mitochondrial morphology in dermal axons was also evaluated with the mitochondrial marker Oxphos ComplexIV Subunit I (COXI) and with PGP9.5. Tissue fixed with glutaraldehyde was processed to Epon, sectioned on an ultramicrotome, and examined in an electron microscope. Further details of these methods are described in Online Resource 1: Supplemental Methods.

### Experimental design and statistical analysis

For in vivo studies, we compared axonal pathologies in hindpaws injected with capsaicin and control solution (Vehicle) from at least 3 mice. Axonal swellings and mitochondrial length in DRG cultures treated with either capsaicin or control solution (DMSO) were determined from at least three replicates. Results with under- and overexpression of Miro or Drp1, GCaMP3 and TMRM intensity were obtained from at least three replicates. Statistical analyses, including evaluations of normality, were conducted with Prism 3.0 (GraphPad Software, La Jolla, CA). For nonparametric Mann–Whitney tests and Kruskal–Wallis tests with Dunn’s multiple comparisons, values are presented as median + interquartile range. For parametric unpaired *t* tests and one-way ANOVAs with Bonferroni corrections, values are presented as mean + standard deviation (SD). Comparisons of mitochondrial length between capsaicin and control groups were performed with a chi-square test. Correlations between dermal nerve density with β-tubulin III and PGP9.5 staining and length of mitochondria and axonal swellings were performed with Pearson’s correlation tests and Spearman’s correlation tests, respectively.

## Results

### Capsaicin induces axonal ovoid formation and axonal loss in vivo and in vitro

**Fig. 1:**
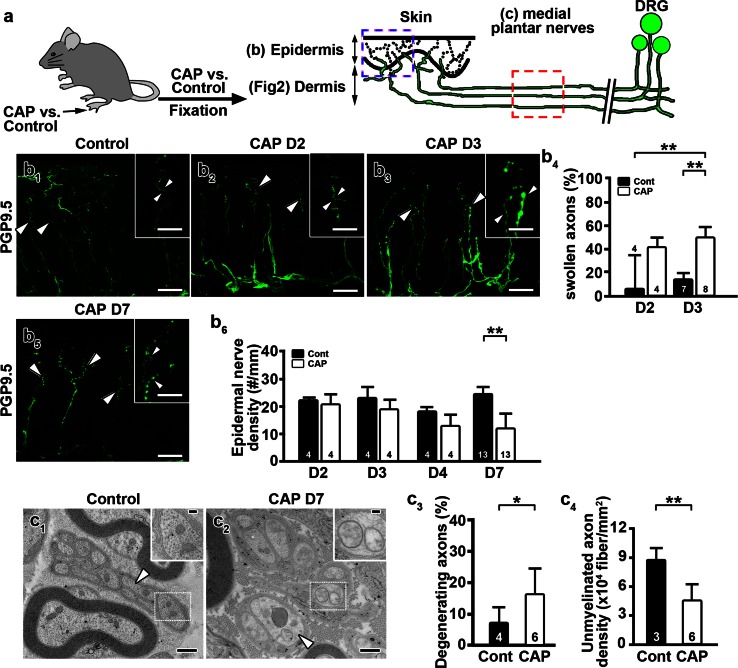
Capsaicin treatment induces axonal degeneration in vivo. **a** Male ICR mice received daily hindpaw intradermal injections of capsaicin (CAP) or vehicle (control) solutions. Unmyelinated axons in the medial plantar nerves (*red dashed box*) and their nerve terminals in the dermis and epidermis (*blue dashed box*) were examined with protein gene product 9.5 (PGP9.5) immunohistochemistry and electron microscopy. *DRG* dorsal root ganglia. **b** Axonal pathologies in epidermal nerves after daily capsaicin injections. **b**_**1**_ Epidermal nerve terminals were evenly distributed in controls (**b**_**1**_ *inset*, *arrowheads*). Following 2 (**b**_**2**_) and 3 (**b**_**3**_) daily capsaicin injections, swelling of epidermal axons was significantly increased (**b**_**4**_). Epidermal nerve density (**b**_**5**_) was significantly reduced (**b**_**6**_) after 7 daily capsaicin (CAP D7) injections (**P < 0.01 by Kruskal–Wallis test and Dunn’s multiple comparison test). *D* day. **c** Electron microscopic images demonstrate unmyelinated axonal degeneration in medial plantar nerves after 7 daily capsaicin injections. Compared to control nerves, which displayed normal axoplasm (**c**_**1**_), unmyelinated axons treated with capsaicin displayed characteristics of degeneration including swollen mitochondria with reduced cristae (**c**_**2**_, *inset*) and pale axoplasm (**c**_**2**_, *arrowhead*). Degenerating axons were increased (**c**_**3**_) and axonal density was decreased (**c**_**4**_) in capsaicin-injected nerves (*P < 0.05, **P < 0.01 by Mann–Whitney test and unpaired *t* test). The numbers of subjects are shown in the *bar graphs*. *Scale Bars* **b**_**1**_–**b**_**3**_, **b**_**5**_: 25 μm; **b**_**1**_ **–b**_**3**_, **b**_**5**_ insets 10 μm; **c**_**1**_ **, c**_**2**_ 500 nm; **c**_**1**_ **, c**_**2**_ insets 100 nm

**Fig. 2:**
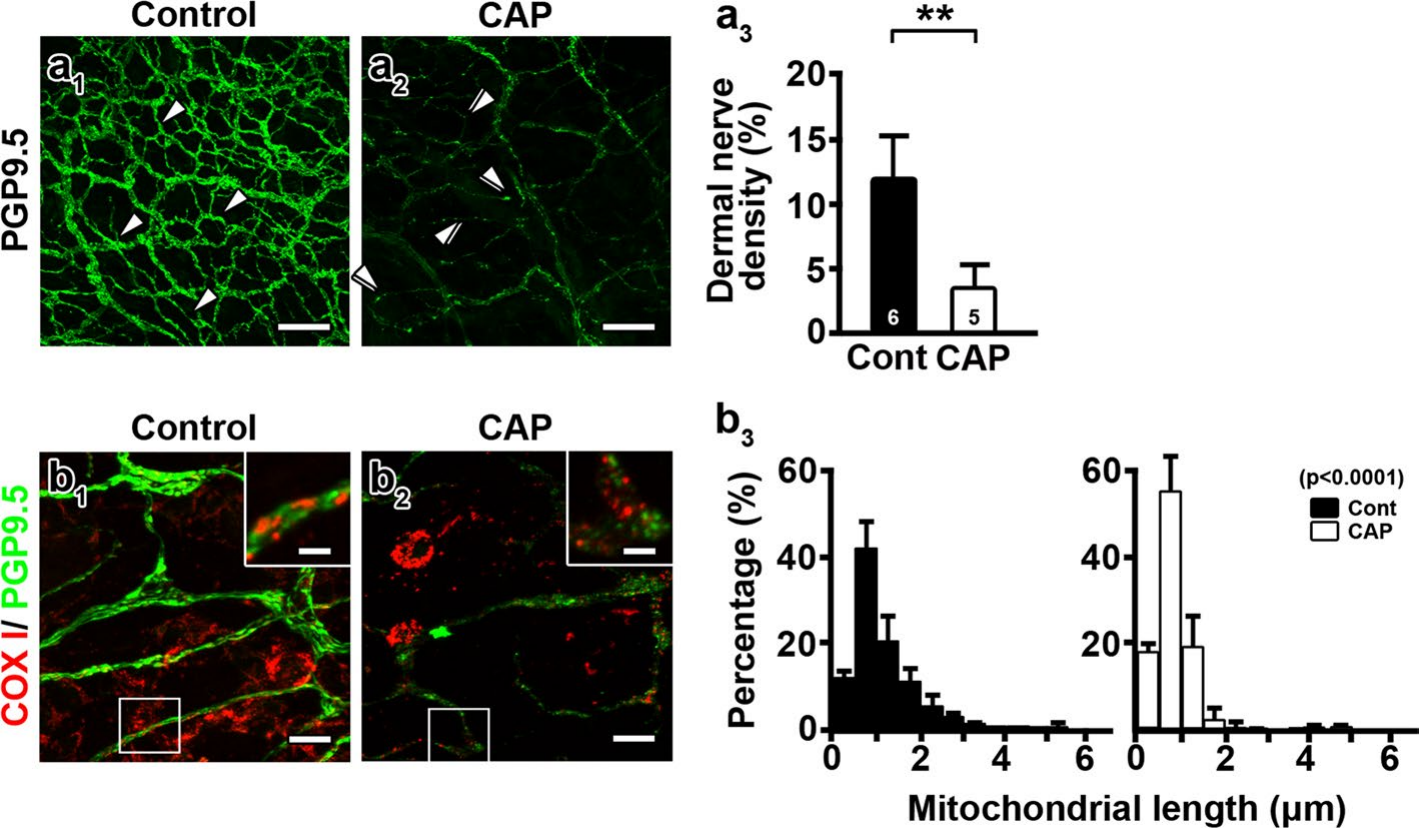
Capsaicin shortens mitochondria and induces axonal degeneration. Dermal axons were examined with protein gene product 9.5 (PGP9.5, *green*) and the mitochondrial marker, Oxphos ComplexIV Subunit I (COXI, *red*) immunohistochemistry. **a**_**1**_, **a**_**2**_ Confocal analyses detected decreased abundance of the dermal nerve plexus after 7 daily capsaicin injections (CAP) (**a**_**2**_) compared to controls (**a**_**1**_, Cont). Dermal nerves were continuous (**a**_**1**_, *arrowheads*) in controls. Axons became beaded (**a**_**2**_, *double arrowheads*) and reduced in number following capsaicin injections (**a**_**3**_) (**P < 0.01 by Mann–Whitney test). The numbers of subjects are shown in the *bar graphs*. **b**_**1**_, **b**_**2**_ COXI-positive mitochondria (COXI) in PGP9.5-positive dermal nerves (PGP9.5) were shorter following capsaicin treatment (**b**_**2**_, *inset*) compared to control nerves (**b**_**1**_, *inset*). **b**_**3**_ Histograms show reduced mitochondrial length after capsaicin treatment (*χ* ^2^ = 144.3, *P* < 0.0001 by chi-square test). Mitochondria from four subjects were pooled. *Scale bars* **a**_**1**_ **, a**_**2**_ 50 μm; **b**_**1**_ **, b**_**2**_ 10 μm; **b**_**1**_ **, b**_**2**_ *insets* 2.5 μm

We investigated capsaicin-induced pathologies in sensory axons in vivo following daily intradermal injections of capsaicin into mouse hindpaws (Fig. 1a). After 3 daily capsaicin injections, there was a significant increase in PGP9.5-positive axonal swellings in the epidermis compared to vehicle-injected hindpaws (Fig. 1b_1_–b_4_). The capsaicin-induced reduction in PGP9.5-positive epidermal nerve density was apparent after 7 daily capsaicin injections (Fig. 1b_5_, b_6_). Loss of PGP9.5 staining was due to axonal loss and not due to reduced PGP9.5 expression in intact axons, as β-tubulin isotype III antibodies produced identical staining patterns as PGP9.5 antibodies (Online Resource 2). Electron microscopic analysis of medial plantar nerves innervating hindpaws detected degenerating axons following 7 daily capsaicin injections (Fig. 1c_1_–c_4_, Online Resource 3). Compared to control unmyelinated axons, which contained intact mitochondria and densely packed neurofilaments (Fig. 1c_1_), unmyelinated axons in capsaicin-treated mice contained swollen mitochondria with reduced cristae and pale axoplasm with decreased neurofilament densities (Fig. 1c_2_). The percentage of degenerating axons was significantly increased (Fig. 1c_3_) and the density of unmyelinated axons was significantly decreased (Fig. 1c_4_) following 7 daily capsaicin injections. Capsaicin-treated Remak bundles contained fewer unmyelinated axons and had an increased percentage of empty collagen pockets, supporting axonal degeneration (Online Resource 4a, b). Dermal axonal density was also decreased following 7 daily injections of capsaicin (Fig. 2a_1_–a_3_). The proportion of mitochondria <1 μm in length was increased significantly in dermal nerves after capsaicin treatment compared to vehicle-treated dermal nerves (Fig. 2b_1_–b_3_). These results indicated that formation of axonal swellings and smaller axonal mitochondria preceded axonal degeneration caused by 7 daily capsaicin hindpaw injections.

**Fig. 3:**
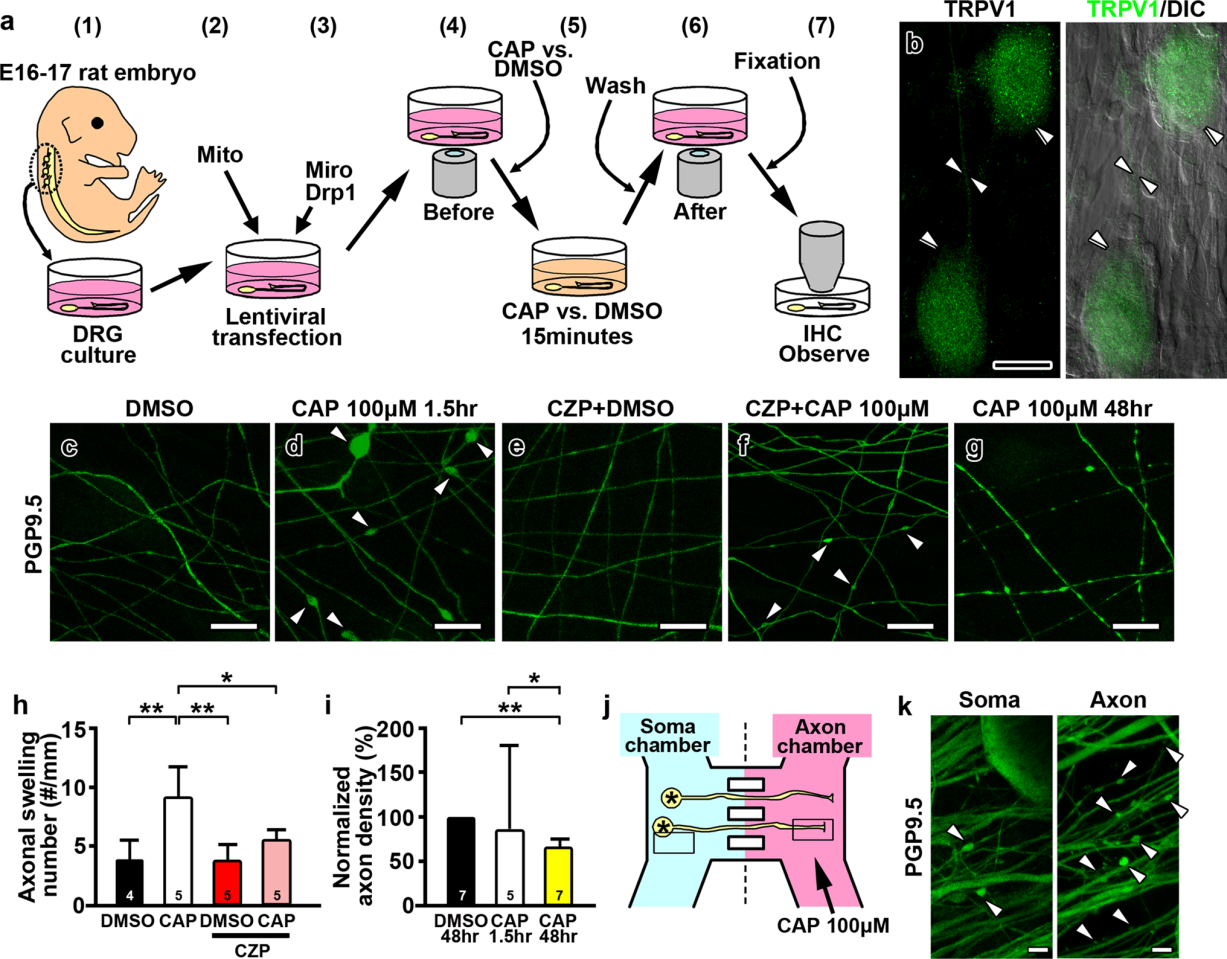
Capsaicin treatment causes axonal ovoid formation and axonal degeneration in vitro. **a** Experimental paradigm of in vitro capsaicin treatment of dorsal root ganglia (DRG) cultures. DRG cultures were prepared from E16 to E17 rat embryos (*1*), transfected with lentiviral vectors carrying mitochondria-targeted fluorescent protein (Mito-Dendra2) (*2*) and then transfected with lentivirus carrying either Miro or Drp1 (*3*). Time-lapse imaging of mitochondria was taken before (*4*), during (*5*), and after treatment with capsaicin (CAP) or vehicle (DMSO) (*6*). Cells were washed and then fixed for immunostaining with PGP9.5. Axonal swellings and mitochondrial morphology were analyzed (*7*). *IHC* immunohistochemistry. **b** DRG neurons (*double arrowheads*) and a representative axon (*arrowheads*) were positive for TRPV1, whereas non-neuronal cells were not. *DIC* differential interference contrast. **c**, **d** Immunostaining of DRG axons for PGP9.5 shows multiple axonal swellings after capsaicin treatment (**d**, *arrowheads*) but not after DMSO treatment (**c**). **e**, **f** Pretreatment with the capsaicin antagonist capsazepine (CZP) decreased axonal ovoid formation upon capsaicin treatment. **g** The number of axons was decreased 48 h after capsaicin treatment. **h** Axonal swellings were increased after capsaicin compared to DMSO treatment and CZP significantly decreased the number of axonal swellings (*P < 0.05; **P < 0.01 by Kruskal–Wallis test and Dunn’s multiple comparison test). **i** *Bar graph* shows decreased axonal density normalized to DMSO 48 h after capsaicin treatment (*P < 0.05; **P < 0.01 by Kruskal–Wallis test and Dunn’s multiple comparison test). **j** In the Xona microfluidic system, DRG neurons were seeded in a soma chamber (*blue*) and extended axons into a separated chamber (*pink*), where capsaicin was applied (*arrow*). **k** Application of capsaicin in the axonal chamber induced axonal swellings (*arrowheads*). Axonal ovoid formation in the soma chamber was less prominent. The numbers of independent replicates are shown in the *bar graphs*. *Scale bars*: **b** 25 μm; **c–g** 10 μm; **k** 5 μm

We next investigated the role of mitochondria in capsaicin-induced axonal pathologies in primary DRG cultures. Figure 3a summarizes the general approach to our in vitro studies. The capsaicin receptor, TRPV1, was detected on the surface of DRG neurons and axons (Fig. 3b). PGP9.5 immunostaining was used to identify unmyelinated axons (Fig. 3c–g) and axons were continuous and uniform in width upon DMSO treatment (Fig. 3c). In contrast, after capsaicin treatment, axons exhibited multiple swellings followed by axonal loss in a dose-dependent and time-dependent manner (Online Resource 5a–h). Based upon this dose response study, 100 μM capsaicin was used for all subsequent experiments. The number of axonal swellings per unit axonal length was significantly increased in the capsaicin group compared to the DMSO group (Fig. 3c, d, h). In addition to axonal ovoid formation, axonal loss in DRG cultures increased significantly 48 h after capsaicin treatment (Fig. 3g, i). Thus, capsaicin-induced axonal pathology and axonal degeneration in vitro were similar to those found in vivo.

The axonal ovoid formation and subsequent axonal loss in the in vitro DRG experiments described above may result from the activation of TRPV1 by capsaicin on axons and/or neuronal cell bodies. Pretreatment with 10 μM capsazepine (CZP), a competitive antagonist of TRPV1, for 10 min prior to the addition of capsaicin significantly decreased axonal ovoid formation (Fig. 3e, f, h) and rescued axonal loss (Online Resource 6a_1_–a_4_), indicating that activation of TRPV1 by capsaicin induced axonal pathology in vitro. To investigate if capsaicin can directly induce axonal pathology and degeneration by binding to axonal TRPV1, we established DRG cultures in a compartmental microfluidic system that isolates the environment of distal axons from that of neuronal cell bodies [69]. 90 min following capsaicin administration (15 min) to the Xona compartment that contained only DRG axons, axonal ovoid formation was more prominent in the axon chamber than in the soma chamber (Fig. 3j, k, axon vs. soma, 4.92 ± 1.73 vs. 1.15 ± 0.52 ovoids/mm, *P* = 0.0226). These results provide direct evidence that capsaicin causes formation of axonal swellings in DRG cultures by activating axonal TRPV1.

### Capsaicin induces mitochondrial fission and reduces mitochondrial transport

**Fig. 4:**
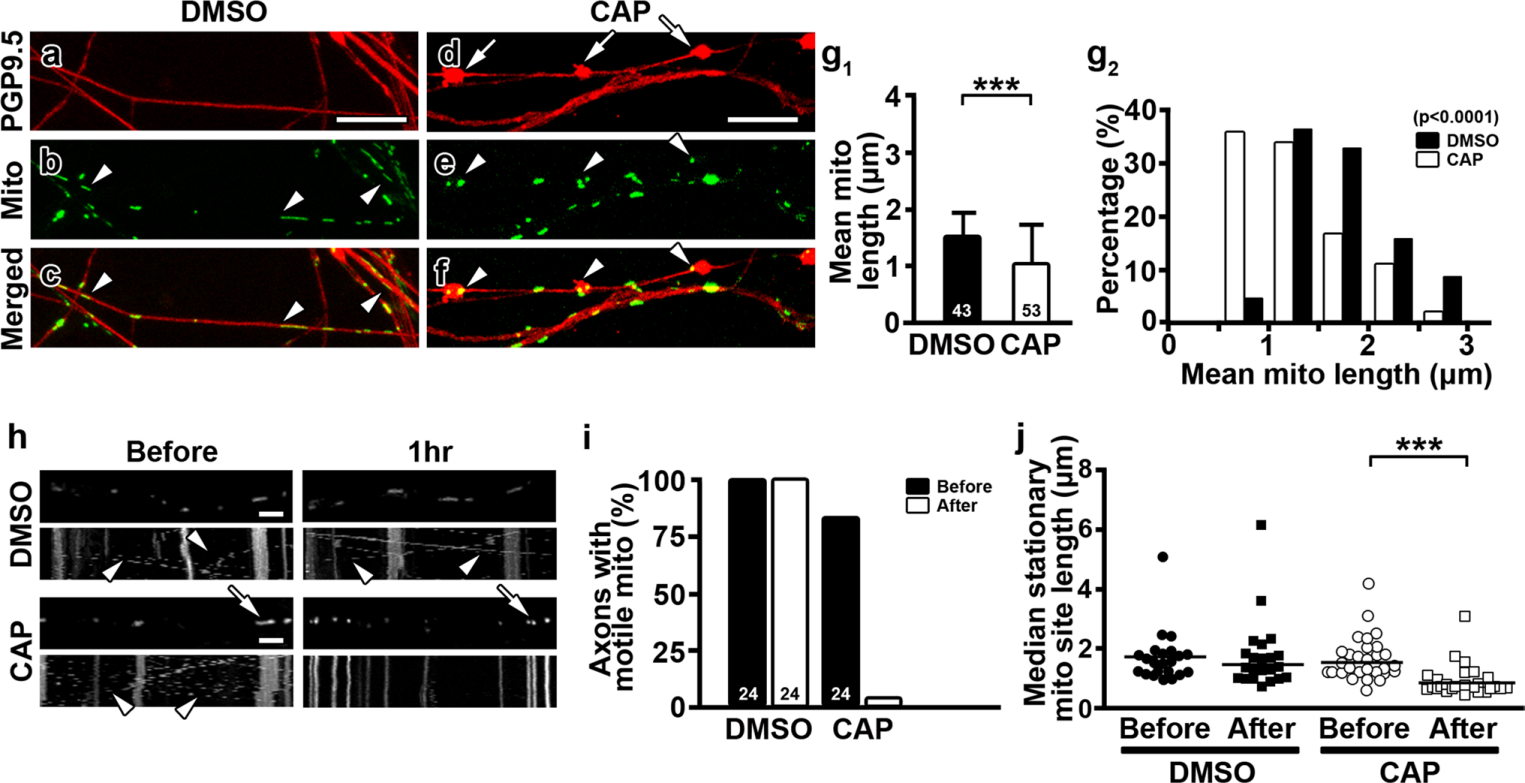
Capsaicin causes fragmentation and reduced motility of axonal mitochondria. **a**–**f** Capsaicin- and DMSO-treated axons were transfected with Mito-Dendra2 (Mito), examined by time-lapse imaging, and then stained with PGP9.5 antibodies. Compared to DMSO-treated nerves (**b**, **c**, *arrowheads*), axonal swellings (**d**, *arrows*) increased and mitochondria became shorter in capsaicin-treated cultures (**e**, **f**, *arrowheads*). **g**_**1**_, **g**_**2**_ Mean mitochondrial length in DRG axons was significantly reduced after capsaicin treatment. When axons were sub-grouped according to their mean mitochondrial length, the proportion of axons with mean mitochondrial length <1 μm was increased upon capsaicin treatment (***P = 0.0001 by Mann–Whitney test. *χ* ^2^ = 34.07, *P* < 0.0001 by chi-square test). **h–j** Representative frames of time-lapse images of axonal mitochondrial transport and resulting kymographs before and 1 h (1 h) after DMSO or capsaicin treatment. The length (*arrows*) and motility (*arrowheads*) of mitochondria were maintained after DMSO but were decreased after capsaicin treatment (***P < 0.0001 by Kruskal–Wallis test and Dunn’s multiple comparison test). The numbers of axons are shown in the *bar graphs*. *Scale bars* **a–f** 10 μm; **h** 5 μm

Abnormal mitochondria in unmyelinated axons of capsaicin-treated medial plantar nerves (Fig. 1c_1_, c_2_ inset) and dermal nerves of hindpaw skin (Fig. 2b_3_) indicate that mitochondrial size and/or distribution might contribute to capsaicin-induced axonal pathology and degeneration. To explore these possibilities, we transfected 3- to 4-week-old DRG neurons with mitochondria-targeted fluorescent proteins (Mito-Dendra2 or Mito-DsRed) and examined the distribution and morphology of axonal mitochondria with time-lapse image recording 2 weeks after the transfection (Fig. 3a). Compared to DMSO-treated cultures (Fig. 4a–c), capsaicin treatment (Fig. 4d–f) significantly reduced the length of axonal mitochondria (Fig. 4g_1_). The proportion of mitochondria <1 μm in length increased upon capsaicin treatment (Fig. 4g_2_). Interestingly, 61.5 % of axonal swellings (67/109) in 34 axons contained mitochondria (Fig. 4a–f) and the mitochondrial density [mitochondrial number/axon length (mm)] within axonal swellings was significantly higher than that in axonal regions without swellings (ovoid portion vs. non-ovoid portion, 400 ± 230.7 vs. 74.18 ± 46.3, *P* < 0.0001). Since reduced mitochondrial length has been associated with decreased fusion or increased fission [11], the decreased mitochondrial length in capsaicin-treated cultures suggested that mitochondrial dynamics were affected upon capsaicin treatment. Thus, we next compared the size of stationary mitochondrial sites and the percentage of axons containing motile mitochondria in axons with time-lapse images taken before and after capsaicin treatment. DMSO treatment had no effect on mitochondrial stationary site size or mitochondrial transport (Fig. 4h–j). In contrast, the percentage of axons with motile mitochondria decreased significantly (Fig. 4i) and the length of mitochondrial stationary sites was reduced (Fig. 4j) upon capsaicin treatment (Fig. 4h). Pretreatment of DRG cultures with the TRPV1 antagonist CZP prior to capsaicin administration rescued mitochondrial motility and mitochondrial stationary site length (Online Resource 6b_1_–b_3_), indicating that capsaicin regulates mitochondrial dynamics through activation of TRPV1. Expression of mitochondria-targeted fluorescent proteins was found in a small number of non-neuronal cells, but mitochondrial size in these cells did not change upon capsaicin treatment (DMSO vs. CAP, 6.24 ± 2.93 vs. 4.76 ± 1.18 μm, *P* = 0.4641). In summary, capsaicin treatment of DRG cultures reduced mitochondrial transport and decreased the length of axonal mitochondrial stationary sites via TRPV1 activation.

**Fig. 5:**
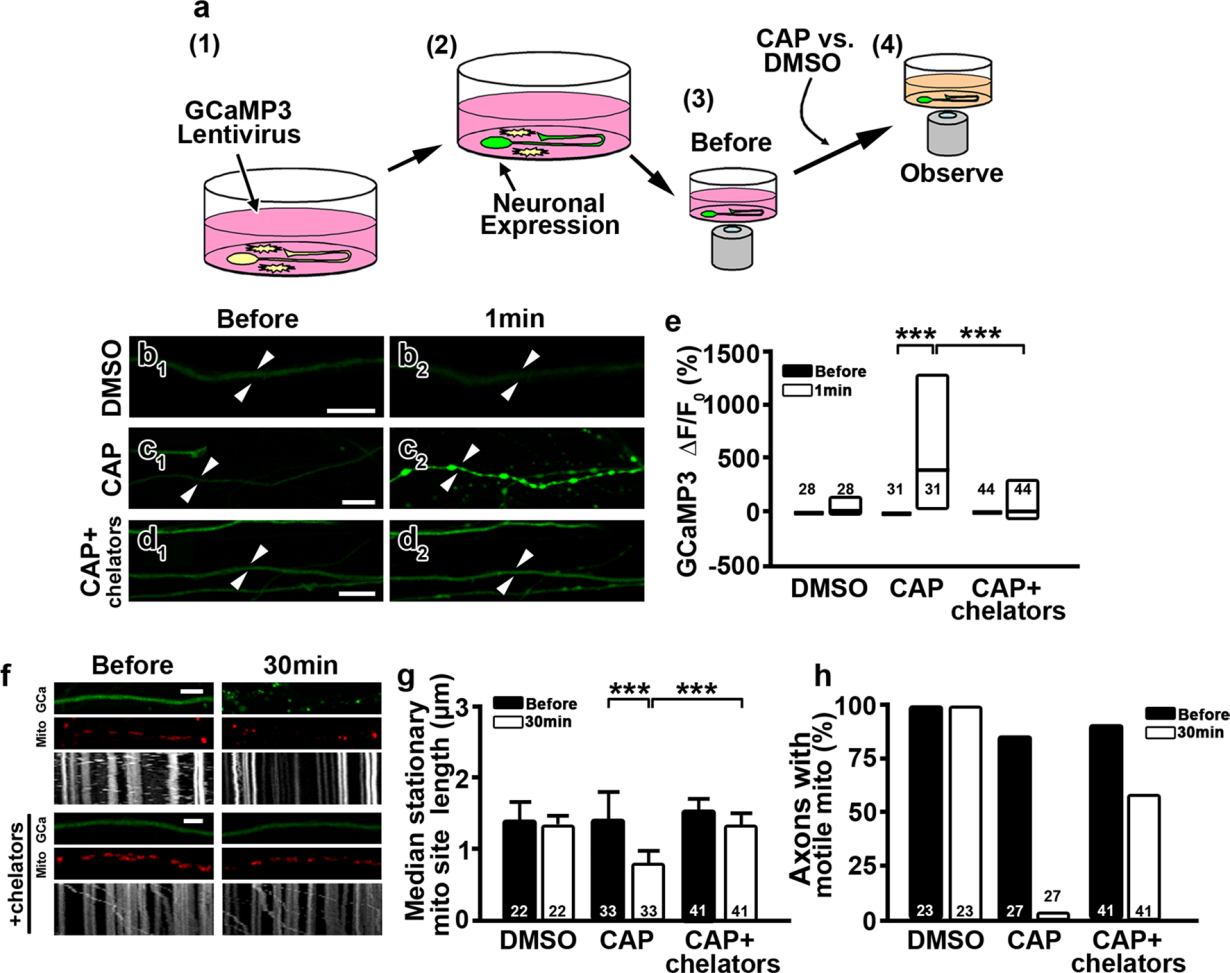
Capsaicin treatment increases axonal Ca^2+^ and fragments axonal mitochondria. **a** Experimental paradigm of axoplasmic Ca^2+^ evaluation following capsaicin treatment. Dorsal root ganglia (DRG) cultures were transfected with a calcium indicator, GCaMP3 (*1*). GCaMP3 activation (fluorescence intensity) (*2*) was observed before (*3*) and during (*4*) capsaicin (CAP) or DMSO treatment. **b–e** Time-lapse images show that axoplasmic fluorescence of GCaMP3 (**b**_**1**_, **c**_**1**_, **d**_**1**_, *arrowheads*) was increased 1 min (1 min) after capsaicin treatment (**c**_**2**_, *arrowheads*), but not after DMSO treatment (**b**_**2**_, *arrowheads*) or when the culture was pretreated with the Ca^2+^ chelators BAPTA-AM and EGTA (CAP + chelators; **d**_**2**_, *arrowheads*). The increase in fluorescent intensity in each axon was normalized to its pretest intensity (Before) (***P < 0.001 by Kruskal–Wallis test and Dunn’s multiple comparison test). Box plots show minimum, median and maximum in **e**. **f–h** Analyses of mitochondrial behavior and GCaMP3 intensity in time-lapse images detected shortened mitochondrial stationary sites (**g**) and reduced mitochondrial transport (**h**) after capsaicin treatment when compared to DMSO or Ca^2+^ chelator treatments (***P < 0.0001 by Kruskal–Wallis test and Dunn’s multiple comparison test). The numbers of axons analyzed are shown in corresponding *bar graphs*. *Scale bars* **b**_**1**_ **–d**_**2**_ 10 μm; **f** 5 μm

Cytosolic Ca^2+^ can regulate mitochondrial dynamics, including mitochondrial motility and mitochondrial stationary site size [26, 46, 60]. To test if axoplasmic Ca^2+^ is increased by capsaicin treatment and is associated with mitochondrial alterations, we doubly transfected DRG neurons with a lentivirus carrying the Ca^2+^ indicator protein GCaMP3 and Mito-DsRed. GCaMP3 contains a single permutated EGFP fused to the calmodulin-binding domain of the myosin light-chain kinase. Thus, an increase in cytosolic Ca^2+^ increases EGFP fluorescence [49]. Changes in axoplasmic Ca^2+^ levels with time-lapse images are indicated by EGFP fluorescence before and immediately after capsaicin exposure (Fig. 5a). The fluorescence intensity of GCaMP3 in DRG axons was generally weak (Fig. 5b–d). The fluorescence intensity was significantly increased in axons treated with capsaicin (Fig. 5c_1_, c_2_, e) but was not altered after the addition of DMSO (Fig. 5b_2_). GCaMP3 was expressed by a small number of non-neuronal cells in DRG cultures, but the fluorescence intensity in those cells did not change upon capsaicin treatment [Before vs. CAP, Δ*F*/*F*_0_ (%) = 0 ± 0 vs. −18.32 ± 48.41 %, *P* = 0.4512], indicating that the effect of capsaicin was neuron specific. To determine whether this Ca^2+^ increase led to altered mitochondrial motility and stationary site size, the Ca^2+^ chelators BAPTA-AM and EGTA were added to the cultures prior to capsaicin treatment. Depletion of free Ca^2+^ in DRG cultures prevented the increase in GCaMP3 fluorescence intensity upon capsaicin treatment (Fig. 5d_1_, d_2_, e). In addition, mitochondrial stationary site sizes were unchanged and 58.5 % of axons maintained motile mitochondria when cultures were pretreated with Ca^2+^ chelators before capsaicin treatment (Fig. 5f–h). These results indicate that a capsaicin-induced increase in axoplasmic Ca^2+^ alters axonal mitochondrial stationary site size and mitochondrial motility.

### Inhibition of mitochondrial fission, but not sustained motility of axonal mitochondria, reduces axonal ovoid formation upon capsaicin treatment

Given that axonal mitochondrial transport was decreased by capsaicin treatment (Fig. 4i), we evaluated the contribution of mitochondrial motility to axonal ovoid formation upon capsaicin treatment by focusing on mitochondrial Rho GTPase (Miro), a mitochondrial outer membrane protein responsible for mitochondrial motility. DRG neurons were transfected with wild-type Miro (MiroWT) or an EF-hand Miro mutant (MiroKK) that prevents a Ca^2+^-induced reduction in axonal mitochondrial transport [46, 60, 78]. For visualizing transfected axons, the *N*′-terminal *myc*-tagged Miro vectors contained a sequence for a reporter fluorescent protein mCherry driven by internal ribosomal entry site (IRES). Upon Miro transfection, axons became positive for mCherry. In addition, *myc*-immunostaining in mCherry-positive axons confirmed the expression of *myc*-tagged Miro in mitochondria positive for Mito-Dendra2 [20] (Online Resource 7a–d). Under time-lapse imaging, the percentage of axons with motile mitochondria was decreased in capsaicin-treated cultures transfected with MiroWT, as observed in control cultures without viral transfection (Online Resource 7e, f). MiroWT overexpression in DRG axons did not alter axonal ovoid formation upon capsaicin treatment (Online Resource 7g–i, m), suggesting the overexpression of MiroWT, which mediated Ca^2+^-induced mitochondrial stopping, did not worsen capsaicin-induced axonal pathology. In contrast, 63.8 % of axons with MiroKK overexpression contained motile mitochondria after capsaicin treatment. However, axonal ovoid formation was not reduced in capsaicin-treated axons that overexpressed MiroKK (Online Resource 7j–l, m). These results indicate that MiroKK overexpression sustains motile mitochondria but did not significantly alter axonal ovoid formation upon capsaicin treatment.

**Fig. 6:**
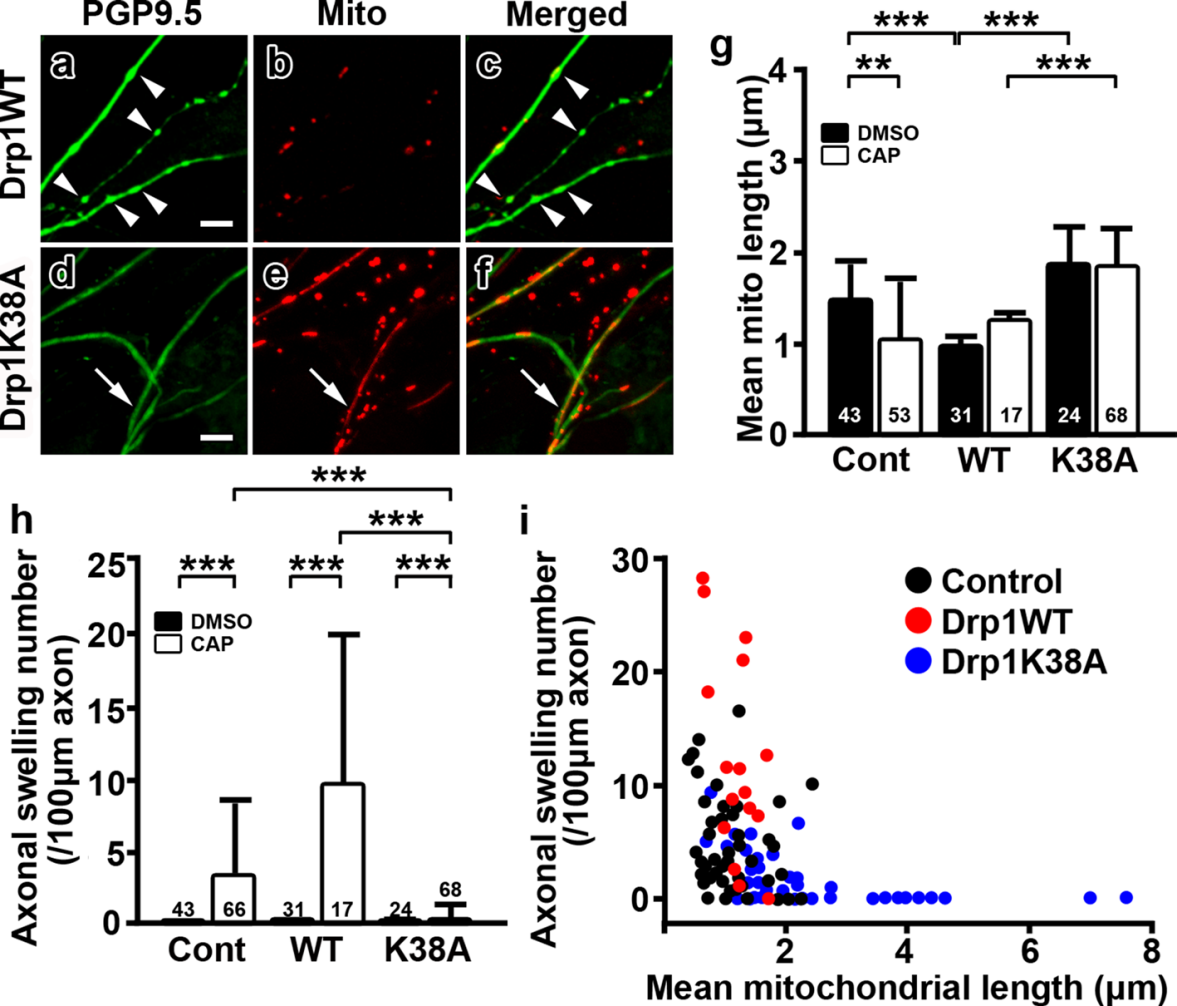
Overexpression of mutant Drp1K38A reduces axonal ovoid formation following capsaicin treatment. **a–f** Confocal images of axonal Mito-Dendra2 (Mito) labeling and PGP9.5 immunostaining after capsaicin treatment (CAP). The axonal swellings and shorter mitochondria (**a–c**, *arrowheads*) that were prominent in axons transfected with wild-type Drp1 (WT) were significantly reduced (**d–f**, *arrows*) in axons transfected with mutant Drp1K38A (K38A). **g** The mean length of axonal mitochondria after capsaicin treatment was significantly increased in Drp1K38A-transfected axons compared to axons transfected with Drp1WT (**P < 0.01; ***P < 0.0001 by Kruskal–Wallis test and Dunn’s multiple comparison test). **h** Capsaicin treatment significantly increased the number of axonal swellings in Drp1WT-transfected axons when compared to mutant Drp1K38A-transfected axons (***P < 0.0001 by Kruskal–Wallis test and Dunn’s multiple comparison test). **i** Mitochondrial length was inversely correlated with the number of axonal swellings in control (*black*), Drp1WT-transfected (*red*) and mutated Drp1K38A-transfected axons (*blue*) (Spearman *r* = −0.6214). The numbers of axons analyzed are shown in the *bar graphs*. *Scale bars* **a–f** 5 μm

**Fig. 7:**
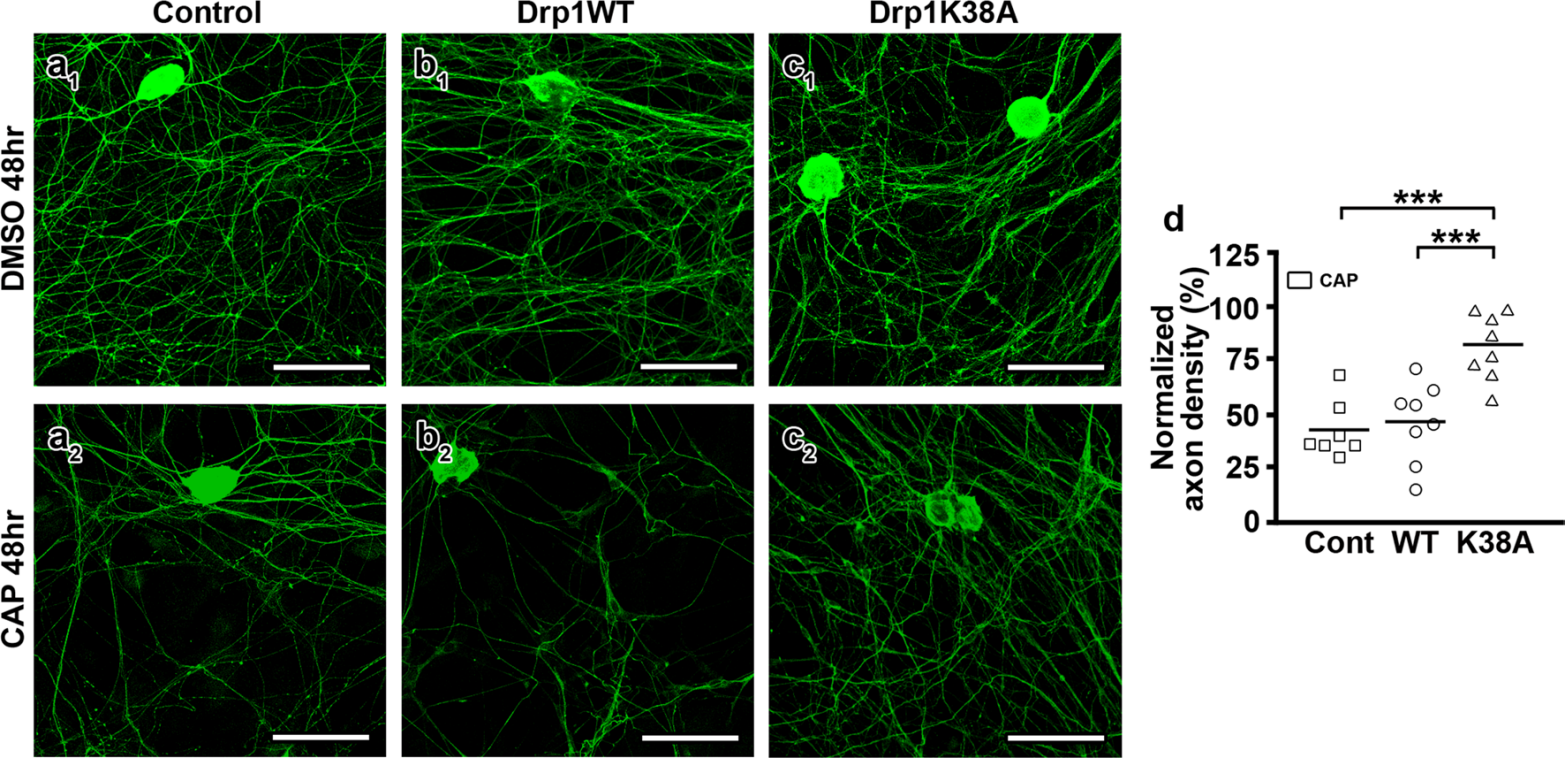
Drp1K38A mutant overexpression improves axonal survival following capsaicin treatment in vitro. **a**_**1**_–**c**_**2**_ Confocal images of control (control; **a**_**1**_, **a**_**2**_), wild-type Drp1-transfected (Drp1WT; **b**_**1**_, **b**_**2**_) and mutated Drp1 (Drp1K38A; **c**_**1**_, **c**_**2**_)-transfected dorsal root ganglia (DRG) cultures immunostained for PGP9.5 48 h after capsaicin (CAP) or DMSO treatment. Capsaicin induced significant axonal loss in control (**a**_**1**_, **a**_**2**_, **d**) and Drp1WT-transfected (**b**_**1**_, **b**_**2**_, **d**) cultures. Axonal density was rescued in Drp1K38A-transfected (**c**_**1**_, **c**_**2**_, **d**) cultures (***P < 0.0001, One-Way ANOVA). The analysis was based on 3–4 randomly chosen fields in each replicate. *Dots* represent number of replicates. *Scale bars* **a**_**1**_ **–c**_**2**_ 100 μm

The decreased size of mitochondrial stationary sites observed in capsaicin-treated axons may reflect an altered balance between mitochondrial fusion and fission. To ask whether preventing mitochondrial fission contributes to axonal pathologies, Drp1 was overexpressed in DRG cultures. When recruited upon increases in cytosolic Ca^2+^, Drp1 mediates mitochondrial fission [10, 19, 26]. Mitochondrial fission in DRG cultures could be modulated by overexpression of wild-type (Drp1WT) or a dominant-negative mutant Drp1 (Drp1K38A), which inhibits Drp1-mediated mitochondrial fission [19, 66]. The Drp1 vectors also contained a mCherry construct to identify Drp1-overexpressing axons. Following capsaicin treatment, axonal mitochondria were shortened (Fig. 6a–c, g) and the number of axonal swellings was increased (Fig. 6h) in cultures with overexpression of Drp1WT. In contrast, capsaicin-treated axons that overexpressed Drp1K38A contained significantly longer mitochondria (Fig. 6d–f, g) and fewer axonal swellings (Fig. 6h) compared to axons transfected with Drp1WT (Fig. 6a–c, g). When the mean of mitochondrial length and the number of swellings in each axon were plotted in a combined dataset from all cultures, mitochondrial length was inversely correlated with the number of axonal swellings (Fig. 6i); i.e., axons with longer mitochondria contained fewer axonal swellings. Furthermore, DRG cultures overexpressing Drp1K38A retained more axons 48 h after capsaicin treatment (Fig. 7c_1_, c_2_, d) compared to DRG cultures without transfection or with Drp1WT overexpression (Fig. 7a, b, d). Taken together, these results demonstrate that inhibition of mitochondrial fission by Drp1K38A resulted in sustained mitochondrial length, reduced axonal ovoid formation, and less axonal degeneration upon capsaicin treatment.

### Inhibited fission of axonal mitochondria retains mitochondrial membrane potential upon capsaicin treatment

**Fig. 8:**
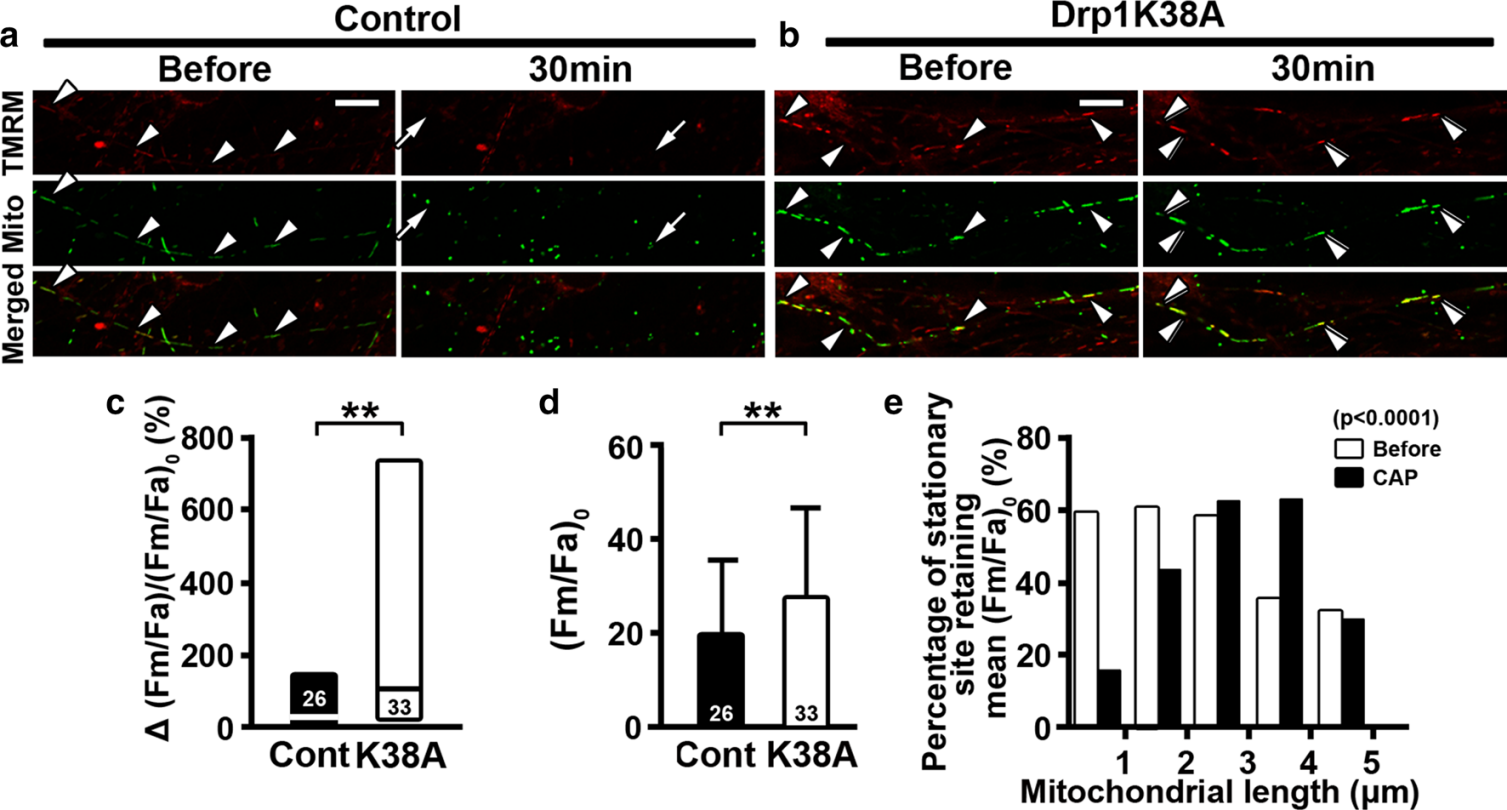
Mutant Drp1K38A overexpression retains mitochondrial membrane potential upon capsaicin treatment. **a, b** Time-lapse images of axonal mitochondria labeled with Mito-Dendra2 (Mito) and stained with TMRM for mitochondrial membrane potential. TMRM labeling co-localized with axonal mitochondria (*arrowheads*). While mitochondria in control (control, Cont) lost TMRM intensity after capsaicin treatment (CAP) (*arrows*), TMRM intensity was retained in Drp1K38A-transfected dorsal root ganglia (DRG) cultures (K38A) (*double arrowheads*). **c** *Bar graphs* show that K38A-overexpressed axons significantly retained TMRM intensity after capsaicin compared to control axons (**P < 0.01 by Mann–Whitney test). **d** Mitochondrial TMRM intensity was significantly increased in axons transfected with mutated Drp1K38A before capsaicin treatment (**P < 0.01 by Mann–Whitney test). **e** Stationary mitochondrial sites with Drp1K38A overexpression sub-grouped according to their lengths were plotted against the percentage of mitochondria with their TMRM intensity exceeding average (Fm/Fa)_0_. *Bar graphs* show that axonal mitochondria of 2–4 μm in length retained membrane potential in K38A-overexpressed axons after capsaicin treatment (*χ* ^2^ = 33.69, *P* < 0.0001 by chi-square test). The numbers of axons in **c** and mitochondrial stationary sites in **d** are shown. *Scale bars* **a**, **b** 10 μm

The inverse correlation between mitochondrial length and axonal degeneration upon capsaicin treatment suggests that inhibition of mitochondrial fission reduces axonal ovoid formation and axonal degeneration by retaining functional mitochondria upon axonal injury. To evaluate the effect of Drp1K38A overexpression on mitochondrial function after capsaicin treatment, we stained Mito-Dendra2-transfected cultures with mitochondrial membrane potential indicator tetramethylrhodamine methyl ester (TMRM). TMRM ubiquitously stained mitochondria in all cell types in DRG cultures (Fig. 8a, b), but mitochondrial TMRM intensity did not change after capsaicin treatment in non-neuronal cells where mitochondria were not labeled with Mito-Dendra2 [Before vs. CAP (Fm/Fc)_0_ = 163.9 ± 211.8 vs. (Fm/Fc)_cap_ = 179.1 ± 216.2, *P* = 0.1748]. Therefore, using Xona platforms, we evaluated TMRM fluorescence within axonal stationary sites labeled with Mito-Dendra2 (Fig. 8a, b). Although TMRM intensity of mitochondria in cultures with Drp1K38A overexpression was significantly higher before capsaicin treatment compared to control cultures (Fig. 8d), there was no correlation between stationary site length and TMRM intensity under basal conditions. While axonal mitochondria in control cultures became shortened and lost TMRM fluorescence upon capsaicin treatment, axonal mitochondria in cultures with Drp1K38A overexpression retained their length and TMRM fluorescence after capsaicin treatment (Fig. 8a–c). Importantly, upon capsaicin treatment, mitochondrial length was correlated with TMRM intensity in axons that overexpressed Drp1K38A; 63 % of mitochondria with a length between 2 and 4 μm retained TMRM intensity after capsaicin treatment (Fig. 8e). These findings support the concept that inhibition of mitochondrial fission by Drp1K38A overexpression retains axonal mitochondrial membrane potential and contributes to axonal survival upon capsaicin treatment.

## Discussion

The present study investigated how activation of the TRPV1 receptor causes degeneration of sensory axonal terminals. We show that capsaicin activation of axonal TRPV1 increases axoplasmic Ca^2+^ and decreases (1) the size of mitochondrial stationary sites, (2) the percentage of axons with motile mitochondria, and (3) mitochondrial membrane potentials. These alterations in axoplasm paralleled axonal ovoid formation and preceded axonal degeneration. Rescue of mitochondrial transport did not rescue axonal ovoid formation or axonal degeneration. Inhibition of mitochondrial fission, however, increased mitochondrial stationary site sizes, reduced axonal ovoid formation and axonal degeneration, and maintained mitochondrial membrane potentials in capsaicin-treated nerves. These results establish a pivotal role of mitochondrial dynamics during axonal ovoid formation and degeneration, and provide evidence that modulation of mitochondrial dynamics can reduce axonal degeneration caused by capsaicin.

In this study, we demonstrate axonal ovoid formation as a pathologic intermediate preceding capsaicin-induced axonal degeneration. Formation of axonal swellings is thought to predict subsequent axonal degeneration in mammals [29, 39, 42]. In the current report, axonal swellings formed upon capsaicin treatment in a dose-dependent manner followed by axonal loss in vivo and in vitro. In addition, reduced axonal ovoid formation paralleled reduced axonal loss. Based on these findings, axonal ovoid formation may be regarded as an early indicator of axonal degeneration.

This report focused on the effects of capsaicin on mitochondrial integrity and axonal degeneration. In addition to increasing axonal Ca^2+^, capsaicin also affects non-neuronal cells, including mast cells [8, 58], which may induce neurogenic inflammation and negatively influence axonal integrity. While this issue requires further investigation in vivo, mast cells are not present in DRG cultures and will not influence the outcomes of capsaicin-induced axonal degeneration.

Local injections of capsaicin in the mouse hindpaw reduced axonal mitochondrial length, increased axonal ovoid formation, and increased distal sensory axonal degeneration. To explore the role of mitochondrial dynamics during capsaicin-induced axonal degeneration, we established an in vitro model which mimics the axonal pathologies in vivo. An important question is whether capsaicin-induced axonal pathology in vitro mimics capsaicin-induced axonal pathology in vivo. TRPV1 was detected on DRG axons in vitro and capsaicin induced axonal swellings and axonal degeneration in a dose-dependent manner. Using the Xona microfluidic culture platform, we established that capsaicin induced axonal ovoid formation by activation of TRPV1 on axons that were segregated from neuronal cell bodies. Inhibition of capsaicin activation of axonal TRPV1 with the capsaicin antagonist CZP significantly reduced axonal swellings, mitochondrial stationary site shortening and axonal degeneration in our in vitro system. Similarities between basic aspects of capsaicin-induced axonal pathology in vivo and in vitro support the use of our in vitro system to study mitochondrial dynamics with confocal imaging.

This current report provides links between mitochondrial dynamics and the pre-degeneration status of nerve fibers, suggesting that altered mitochondrial dynamics, especially shortening of mitochondria, play key roles in axonal ovoid formation. Post-translational modifications of Drp1 and subsequent activation of signaling cascades, including calcineurin and Ca^2+^/calmodulin-dependent protein kinase I alpha (CaMKIalpha), regulates Ca^2+^-mediated mitochondrial fission [10, 12, 14, 26]. Drp1 is essential for normal brain development and total ablation of Drp1 is embryonic lethal and causes severe neurodegeneration [33, 76]. Aberrant activation of Drp1 is thought to trigger neuronal apoptosis in several neurodegenerative diseases, including Huntington’s disease and Alzheimer’s disease, and may be related to the mitochondrial release of the pro-apoptotic molecule Bax [14, 25]. While previous studies focused on Drp1 in neuronal cell bodies and its role in neuronal death, we investigated the role of Drp1 during axonal degeneration caused by receptor-mediated Ca^2+^ influx. We used a dominant interfering mutant Drp1K38A, which prevents shortening of axonal mitochondria by inhibiting mitochondrial fission [4, 55, 74]. Our data demonstrate an inverse correlation between mitochondrial length and axonal degeneration. Sustained mitochondrial length correlated with increased mitochondrial membrane potentials and reduced axonal pathology caused by capsaicin treatment. Mutant Drp1K38A overexpression, which reduces mitochondrial fission, increased mitochondrial length and significantly reduced capsaicin-induced axonal loss from ~50 to ~15 % (Fig. 7d). Overexpression of wild-type Drp1 reduced mitochondrial length (Fig. 6g) but did not change axonal survival (Fig. 7d) following capsaicin treatment, supporting previous studies reporting that Drp1 overexpression can increase fragmentation of mitochondria [40, 68] but does not change cell viability, even at high levels of expression [68]. On the other hand, mutant Drp1 will compete with wild-type Drp1 and reduce mitochondrial fission and axonal degeneration following capsaicin treatment, resulting in increased mitochondrial length (Fig. 6g; [44]).

In our study, shorter stationary mitochondrial sites produced by excessive fission disrupted axonal integrity, whereas longer stationary sites produced by preventing fission decreased axonal damage upon capsaicin treatment. This implies that longer mitochondrial stationary sites are an indication of better mitochondrial function. Capsaicin reduced the size of mitochondria and decreased TMRM mitochondrial intensity in control cultures; however, when mutant Drp1 was overexpressed in capsaicin-treated axons, mitochondrial length and TMRM mitochondrial potential were not decreased to the same extent as in capsaicin-treated control cultures (Fig. 8e). Longer stationary sites may have a greater capacity for buffering axoplasmic Ca^2+^ increases upon capsaicin treatment. Longer mitochondria may retain the ability to produce more ATP during and/or following capsaicin treatment and thereby decrease axonal damage. Mitochondrial fusion and elongation are beneficial under starvation and stress caused by UV irradiation and this has been attributed to increased ATP production [24, 57, 72]. In addition, mitochondrial swelling and reduced mitochondrial cristae are indicative of mitochondrial dysfunction and may contribute to decreased axonal ATP production and reduced axonal survival [50, 54, 60]. The dynamin-related proteins, including OPA1 and Drp1, have been shown to regulate changes in cristae during apoptosis [21, 22]. Inhibition of mitochondrial fission by mutant Drp1 may help retain cristae morphology and mitochondrial functions upon capsaicin treatment. A fusion-prone shift in the balance between mitochondrial fusion and fission by temporarily suppressing Drp1 function or its associated regulators may provide a therapeutic target for acute axonal injury caused by Ca^2+^ overload and energy deficits [34].

Due to significant mitochondrial transport reduction induced by capsaicin treatment, we asked whether changing mitochondrial motility by overexpressing MiroWT or MiroKK would alter axonal ovoid formation induced by capsaicin. Movement of mitochondria relies on their binding to the cytoskeleton through adaptors and Miro is a key regulator in Ca^2+^-mediated mitochondrial stopping. MiroWT overexpression did not rescue or worsen axonal ovoid formation. On the other hand, overexpression of MiroKK sustained mitochondrial motility but did not rescue axonal ovoid formation upon capsaicin treatment. While it is likely that chronic alterations in mitochondrial transport can have a negative impact on mitochondria turnover and axonal viability, our data support the concept that acute changes in mitochondrial transport do not affect axonal viability.

Our results establish a key role of mitochondrial size in capsaicin-induced axonal degeneration. Other mechanisms dependent on mitochondria, including autophagy/mitophagy, may also have an impact on capsaicin-induced axonal degeneration. Uncontrolled autophagy/mitophagy has been shown to contribute to axonal degeneration in neurodegenerative diseases [79, 80]. We did not observe autophagy in electron microscopic images of capsaicin-treated nerves in vivo nor did we observe autophagosomes in capsaicin-treated dermal nerves with Light Chain 3 (LC3) immunostaining, which is an indicator of autophagy (Online Resource 8). It is possible that axoplasmic Ca^2+^ increases induced by capsaicin may cause autophagy and/or impair axonal integrity [30] at time points not analyzed in the current study. Further studies, including western blotting for LC3 and live imaging for GFP-LC3, are needed to evaluate autophagy/mitophagy in capsaicin-induced axonal loss.

**Fig. 9:**
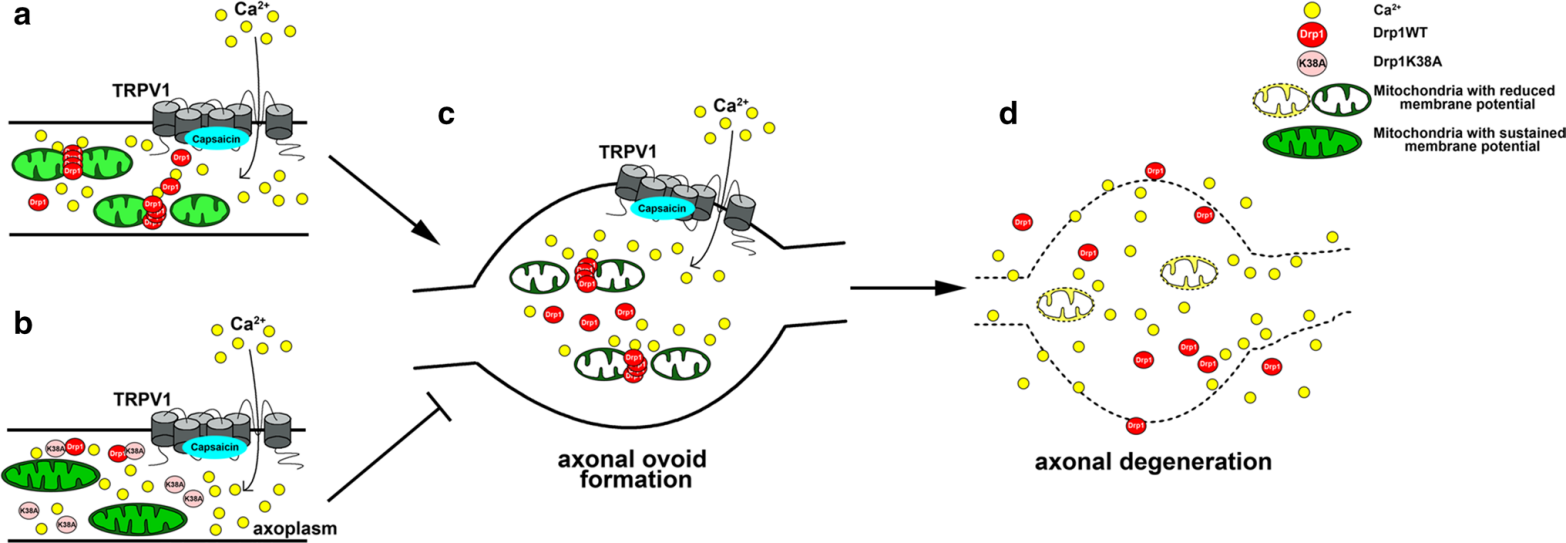
Proposed model for the role of mitochondrial fission in capsaicin-induced axonal degeneration. **a** Activation of TRPV1 increases axoplasmic Ca^2+^, induces Drp1-mediated mitochondrial fission, reduces mitochondrial membrane potential, and increases axonal swellings (**c**) and axonal degeneration (**d**). **b** When mitochondrial fission is prevented by mutant Drp1K38A overexpression, axonal mitochondria retain normal lengths and sustain membrane potentials. This correlates with decreased axonal ovoid formation and axonal degeneration upon capsaicin treatment

This study highlights the importance of mitochondrial dynamics in maintaining axonal integrity. Axonal degeneration causes functional impairments and often precedes neuronal cell death. We evaluated mechanisms of axonal degeneration induced by capsaicin, an analgesic which has been shown to induce distal axonal degeneration. Capsaicin activates TRPV1. When activated, TRPV1 mediates a rapid increase in axoplasmic Ca^2+^ [9, 64]. Our results demonstrate that capsaicin induces axonal degeneration through Ca^2+^-dependent alterations in mitochondrial dynamics. The rescue of axonal survival by inhibition of mitochondrial fission provides further evidence that mitochondria are actively involved in axonal degeneration (Fig. 9a–d). Our model shares important aspects of the axonal swellings and dying-back axonal degeneration described in genetic neurodegenerative diseases [17, 28], including hereditary spastic paraplegia (HSP) [6] and Charcot–Marie–Tooth disease [53]. HSP is caused by a variety of gene mutations, including genes specific for myelin (proteolipid protein), axonal transport (kinesis), smooth endoplasmic reticulum (reticulin) and mitochondria (heat shock protein 60, paraplegin, and mitofusin2) [6, 17, 48, 51, 81]. Despite this diversity in mutated genes, the pathological hallmark of HSP is a dying-back axonopathy that is mediated in part by chronic increases in axoplasmic Ca^2+^. Defects in mitochondrial genes or functions are also documented in non-hereditary chronic neurodegenerative diseases [38, 59, 63]. Diabetic sensory neuropathies, with significant distal axonal loss and reduced mitochondrial functions in both humans [35, 42] and animal models [15, 65], add additional support for the interdependence between mitochondria and axonal integrity. In contrast to chronic neurodegenerative diseases, capsaicin acutely induces axonal degeneration by increasing axoplasmic Ca^2+^. Given the difficulties in manipulating mitochondrial changes in chronic models of axonal degeneration, this report describes an acute system which can be tested and easily regulated in vitro. The capsaicin model shares fundamental aspects of chronic axonal degeneration.

In conclusion, we demonstrate a critical role for mitochondrial dynamics in axonal ovoid formation and subsequent axonal degeneration by altering fusion and fission. Axonal ovoid formation induced by increased axoplasmic Ca^2+^ can be modulated by a Drp1-mediated pathway, which impacts axonal survival. Thus, our capsaicin model is simple, easily regulated, and provides a paradigm to rescue axons upon cationic overload in neurodegenerative diseases.

## Electronic supplementary material

Below is the link to the electronic supplementary material.

Supplementary material 1 (DOCX 3952 kb)

## Abbreviations

BAPTA-AM: 1,2-bis-(o-Aminophenoxy)-ethane-*N*,*N*,*N*′,*N*′-tetraacetic acid, tetraacetoxymethyl ester
CAP: Capsaicin
CZP: Capsazepine
DRG: Dorsal root ganglia
Drp1: Dynamin-related protein 1
Miro: Mitochondrial Rho GTPase
PGP9.5: Protein gene product 9.5
COXI: Oxphos ComplexIV Subunit I
TMRM: Tetramethylrhodamine methyl ester
TRPV1: Transient receptor potential vanilloid receptor 1
