# Supplementary material for: Mitochondrial fission augments capsaicin-induced axonal degeneration
Source: Acta Neuropathol. 2014 Oct 17;129(1):81–96. doi: 10.1007/s00401-014-1354-3 (PMC4282704; doi:10.1007/s00401-014-1354-3)
Supplement: Supplementary file 1 — Supplementary material 1 (DOCX 3952 kb) [file 401_2014_1354_MOESM1_ESM.docx]

Submitted to Acta Neuropathologica

**Title: Mitochondrial Fission Augments Capsaicin-induced Axonal Degeneration**

**Authors:** Hao Chiang^1,2^, Nobuhiko Ohno^2^, Yu-Lin Hsieh^1^, Don J Mahad^2^, Shin Kikuchi^2^, Hitoshi Komuro^2^, Sung-Tsang Hsieh^1,3^, and Bruce D. Trapp^2^

**Affiliations:** ^1^Department of Anatomy and Cell Biology, National Taiwan University College of Medicine, Taipei, 10051, Taiwan; ^2^Department of Neurosciences, Lerner Research Institute, Cleveland Clinic, Cleveland, OH 44195**;** ^3^Department of Neurology, National Taiwan University Hospital, Taipei, 10002, Taiwan

**Corresponding authors:** Dr. Bruce D. Trapp ([trappb@ccf.org](mailto:trappb@ccf.org)); Dr. Sung-Tsang Hsieh ([shsieh@ntu.edu.tw](mailto:shsieh@ntu.edu.tw))

**Online Resource 1: Supplemental Methods**

Embryonic primary DRG culture

Thoracic and lumbar DRG from E16-17 Sprague-Dawley rats were dissected and dissociated by chemical digestion in 0.25% trypsin in L-15 medium (Sigma) for 15 minutes at 37^o^C. Following mechanical trituration in flame polished Pasteur pipettes, dissociated cells were washed with L-15 medium and resuspended in Neurobasal medium (Invitrogen, Calsbad, CA) containing 1X B-27 supplement (Invitrogen), 100ng/ml nerve growth factor (BD Biosciences, San Jose, CA), 1X Glutamax (Invitrogen) and Penicillin/Streptomycin (Invitrogen). Dissociated cells were plated on glass-bottom dishes (MatTek, Ashland, MA) or glass coverslips with Xona microfluidic slide systems (Xona Microfluidics, Temecula, CA) coated with 5% matrigel (BD Biosciences). Cultures were maintained at 37^o^C, 5% CO_2_ and half the medium was replenished every 2-3 days.

Lentiviral transfection

pLenti6/V5 vectors (Invitrogen) containing GCaMP3 (Addgene, Cambridge, MA) [[12](#_ENREF_12)] or mitochondrial-targeted DsRed2 (Mito-DsRed, Clontech, Mountain View, CA) sequences for detecting axoplasmic Ca^2+^ levels and labeling of mitochondria, respectively, were generated as described previously [[7](#_ENREF_7),[10](#_ENREF_10)]. Additional molecules included (1) Mito-Dendra2 as a mitochondrial reporter by adding a cleavable mitochondrial-targeting sequence from cytochrome *c* oxidase subunit VIII to the N-terminus of Dendra2 (Clontech) [[15](#_ENREF_15)]; (2) mitochondrial Rho GTPase (Miro), a mitochondrial outer membrane protein responsible for Ca^2+^-mediate mitochondrial stopping (wild-type and mutant Miro sequences were generous gifts from Dr. Pontus Aspentröm [[3](#_ENREF_3)]); and (3) Drp1 for evaluating the role of mitochondrial fission in axonal integrity. Wild-type Drp1 was obtained from Drp1-GFP as a generous gift from Dr. Richard Youle [[2](#_ENREF_2)]. The Drp1K38A mutant, which inhibits mitochondrial fission, was generated by QuikChange II XL Site-directed Mutagenesis Kits (Agilent Technologies, Palo Alto, CA) using primers gaacgcagagcagcggagcgagctcagtgctagaaag and tttctagcactgagctcgctccgctgctctgcgttc. Miro/Drp1 and Mito-Dendra2 sequences were subcloned into pLVX-IRES-mCherry (Clontech) and pLenti6/V5 vectors with polymerase chain reaction (PCR) methods. All constructs were sequenced to assure correct sequences. Lentivirus with pLVX and pLenti6/V5 vectors were generated in 293T cells following published protocols [[10](#_ENREF_10)]. DRG cultures at 3-4 weeks *in vitro* were transfected with Mito-DsRed, Mito-Dendra2 or GCaMP3 and further incubated for 2 weeks prior to confocal analysis and/or fixation. Some of the cultures transfected with Mito-Dendra2 were additionally transfected with either wild-type or mutant Miro or Drp1, 5 to 7 days prior to live imaging analysis and/or fixation. Mitochondrial-targeted lentiviruses preferentially infect DRG neurons and label all axonal mitochondria in transfected cells, as described previously [[7](#_ENREF_7)].

Time-lapse imaging and quantification for mitochondrial dynamics and GCaMP3 intensities

For time-lapse imaging of mitochondrial dynamics, lentiviral-transfected culture dishes were transferred to a Stage Top Incubator (Tokai Hit, Shizuoka-ken, Japan) attached to an inverted laser-scanning confocal microscope (TCS SP5, Leica, Solms, Germany), and maintained at 37^o^C and constant flow of 95% air and 5% CO_2_. Randomly chosen axons positive for Mito-DsRed2, Mito-Dendra2 or GCaMP3 with or without mCherry were imaged using a 20X oil-immersion objective (NA 0.7) at 1024X1024 pixel resolution. Prior to adding capsaicin, mitochondrial images were taken at every 3 seconds for 41 images. Due to the difficulty in maintaining focal planes immediately after capsaicin addition or replenishment of fresh medium, mitochondrial time-lapse images after capsaicin treatment were obtained 15-75 minutes after the medium replenishment.

For GCaMP3 time-lapse recording, DRG cultures seeded in Xona platforms were transferred to a large chamber (Slide-in module standard XL multi S1, Zeiss, Oberkochen, Germany), seated on an inverted time-lapse laser scanning microscope (LSM 780, Zeiss), and maintained at 37^o^C and constant flow of 95% air and 5% CO_2_. Images of randomly chosen distal axons positive for GCaMP3 and Mito-DsRed2 in the axon chamber were taken at every 3 seconds for 41 images before capsaicin addition. Images were taken using a 63X oil-immersion objective (Plan-Apochromat, NA 1.4, Zeiss) at 512X512 pixel resolution. In a set of experiments, DRG cultures were pre-incubated with 10 μM 1,2-bis-(o-aminophenoxy)-ethane-N,N,N',N'-tetraacetic acid, tetraacetoxymethyl ester (BAPTA-AM, Enzo Life Sciences, Farmingdale, NY) for 30 minutes followed by 10 mM EGTA (Sigma) to chelate intracellular and extracellular free calcium, respectively. After capsaicin addition, GCaMP3 images were immediately taken as series of single images. After imaging, Ca^2+^ ionophore 10 μM ionomycin (LKT Lab, St. Paul, MN), dissolved in medium without Ca^2+^ chelators, was added to the culture to equilibrate intracellular and extracellular Ca^2+^ to ensure the decreased GCaMP3 intensity 1 hour after capsaicin addition was not due axonal degeneration.

Kymographs of mitochondria in time-lapse images were produced using ImageJ (National Institute of Health) as described previously [[9](#_ENREF_9)]. Stationary sites were defined as mitochondrial profiles without displacement in the total imaging time-frames. Motile mitochondria were small and moving in both anterograde and retrograde directions. The lengths of individual stationary sites were measured. The median stationary mitochondrial site length was determined for individual axons and included measures from 3-5 mitochondrial stationary sites. Axons with motile mitochondria were counted. To quantify changes of GCaMP3 fluorescent intensity, images were converted to 8 bit grayscale using ImageJ. Mean grey values for individual axons were obtained by averaging 5 images at approximately 30 second intervals. Differences in mean grey values (ΔF) at baseline (F_0_) and upon capsaicin treatment were divided by F_0_ to normalize and obtain ΔF/F_0_.

Time-lapse imaging and analysis for mitochondrial membrane potentials

To evaluate mitochondrial membrane potential in axons, lentiviral-transfected DRG cultures seeded in Xona platforms were stained with tetramethylrhodamine methyl ester (TMRM, Invitrogen) as previously described with some modifications [[14](#_ENREF_14)]. The fluorescent intensity of TMRM inversely indicates mitochondrial membrane potential (∆ψ_m_) [[1](#_ENREF_1),[8](#_ENREF_8)]. Briefly, axonal chambers of Xona platforms were incubated with 20 nM TMRM at 37 ^o^C for 20 minutes. To ensure homogenous staining in the axonal chambers, medium containing TMRM was re-added to one of the axonal chambers every 5 minutes. Axonal chambers were then replenished with 5 nM TMRM and transferred to the incubation chamber on an inverted- time-lapse laser scanning microscope (LSM 780, Zeiss) for time-lapse imaging. Condition settings were identical to those described for GCaMP3 imaging. Serial time-lapse images were taken before and after capsaicin treatment followed by addition of 20 μM proton gradient uncoupler carbonyl cyanide *m*-chlorophenyl hydrazone (CCCP, Sigma) to ensure the dissipation of mitochondrial membrane potential dye was the result of membrane potential depolarization.

Fluorescent intensity of TMRM images in mitochondrial stationary sites was measured with ImageJ as described previously [[14](#_ENREF_14)]. TMRM fluorescent intensity at stationary sites in serial time-lapse images was outlined manually and averaged using ImageJ. Background axoplasmic fluorescence was also determined in a similar-sized region proximal to the stationary sites. To measure the ratio between absolute mitochondrial and axoplasmic fluorescence, fluorescent intensity at stationary sites (F_m_) was first subtracted by 2/3 axoplasmic fluorescence (F_axon_) since the diameter of mitochondria was 1/3 of the diameter of axons. This ratio was determined by electron microscopic analysis of axons maintained *in vitro* for 1 month (Figure S1). Then absolute mitochondrial fluorescence was normalized with 1/3 axoplasmic fluorescence. The ratio of mitochondrial fluorescence to corresponding axoplasmic fluorescence can be determined with the following formula: Fm/Fa=[F_m_-(2/3)F_axon_]/(1/3)F_axon_. Differences in mitochondrial TMRM intensity (ΔFm/Fa)_cap_ at baseline (Fm/Fa)_0_ and upon capsaicin treatment were normalized by F_0_ to obtain ΔF/F_0_.

Axonal pathology

Hindpaw skin and associated medial plantar nerves from the injection sites were resected after transcardial perfusion with 4% paraformaldehyde and used for subsequent analyses. DRG cultures were fixed with 4% paraformaldehyde solution 90 minutes or 48 hours after drug treatment. Epidermal nerves in the hindpaw skin and DRG cultures were immunochemically stained for protein gene product 9.5 (PGP9.5), a cytosolic ubiquitin C-terminal hydrolase enriched in sensory axons, as described previously [[5](#_ENREF_5),[11](#_ENREF_11)]. An axonal structural marker, ß-tubulin isotype III (ß-tubulin III), was also used to confirm axonal integrity. Mitochondrial morphology in dermal axons was evaluated with the mitochondrial marker Oxphos ComplexIV Subunit I (COXI) and with PGP9.5. To obtain dermis, the glabrous skin adjacent to the footpad skin was resected and incubated in an EDTA solution at 37 ^o^C for 30 minutes to remove the epidermis [[13](#_ENREF_13)]. Fixed tissues were then cut on a sliding microtome (Microm, Walldorf, Germany). Prior to the immunostaining, dermal sheets were first incubated in 10 mM EGTA (pH 8.0) antigen retrieval solution and then microwaved for 20 seconds. Primary antibodies were specific for PGP9.5 (UltraClone, Isle of Wight, UK), COXI (Invitrogen), ß-tubulin isotype III (Sigma), LC3B (Cell Signaling, Danvers, MA), and transient receptor potential vanilloid subtype 1 (TRPV1) (Neuromics, Edina, MN). Secondary antibodies were conjugated with Alexa Fluor 488, Alexa Fluor 647, or Cyanine 3 (Cy3) (Jackson ImmunoResearch, Philadelphia, PA). Images were photographed using a confocal microscope and 63X lens (LSM 780, Zeiss).

Epidermal axons were quantified as described previously [[5](#_ENREF_5)]. Epidermal fibers with branching points within the epidermis were counted as a single fiber, whereas those with branching points located in dermis were counted as multiple fibers. The number of epidermal fibers present along the lower margin of stratum corneum defined the epidermal nerve density. Axons with more than 3 terminal axonal varicosities whose diameters were twice that of the stem portion of the axon were defined as swollen axons. Axonal ovoid density and axonal density *in vitro* were analyzed with images taken by confocal microscopy at 200X or 400X magnification (LCS SP5, Leica). Axonal swellings were defined as axonal spheroids which had single or dual axonal connections and were at least twice as wide as adjacent areas of the same axons. Axonal swellings and mitochondrial stationary site size were quantified per axonal length. Axonal density was defined as the total length of axons per unit area (μm/μm^2^). All parameters were measured with ImageJ software. Automated neurite length measurement plugin NeurphologyJ was used to measure total axonal length [[4](#_ENREF_4)].

Transmission electron microscopy

For electron microscopic studies, terminal branches of medial plantar nerves were processed to Epon by standard procedures [[6](#_ENREF_6)]. Images were taken at 8000X magnification in a Hitachi H-7100 (Hitachi, Tokyo, Japan). The percentage of unmyelinated axons undergoing Wallerian-like degeneration per total unmyelinated axons was calculated. For unmyelinated axonal degeneration, the number of normal-appearing unmyelinated axons in the area enclosed by the endoneurium was defined as unmyelinated axonal density (axons/mm^2^).

References

1. Ehrenberg B, Montana V, Wei MD, Wuskell JP, Loew LM (1988) Membrane potential can be determined in individual cells from the nernstian distribution of cationic dyes. Biophys J 53 (5):785-794. doi:10.1016/S0006-3495(88)83158-8

2. Frank S, Gaume B, Bergmann-Leitner ES, Leitner WW, Robert EG, Catez F, Smith CL, Youle RJ (2001) The role of dynamin-related protein 1, a mediator of mitochondrial fission, in apoptosis. Dev Cell 1 (4):515-525. doi:10.1016/S1534-5807(01)00055-7

3. Fransson S, Ruusala A, Aspenstrom P (2006) The atypical Rho GTPases Miro-1 and Miro-2 have essential roles in mitochondrial trafficking. Biochem Biophys Res Commun 344 (2):500-510. doi:10.1016/j.bbrc.2006.03.163

4. Ho SY, Chao CY, Huang HL, Chiu TW, Charoenkwan P, Hwang E (2011) NeurphologyJ: an automatic neuronal morphology quantification method and its application in pharmacological discovery. BMC Bioinformatics 12:230. doi:10.1186/1471-2105-12-230

5. Hsieh ST, Chiang HY, Lin WM (2000) Pathology of nerve terminal degeneration in the skin. J Neuropathol Exp Neurol 59 (4):297-307

6. Hsieh YL, Chiang H, Tseng TJ, Hsieh ST (2008) Enhancement of cutaneous nerve regeneration by 4-methylcatechol in resiniferatoxin-induced neuropathy. J Neuropathol Exp Neurol 67 (2):93-104. doi:10.1097/nen.0b013e3181630bb8

7. Kiryu-Seo S, Ohno N, Kidd GJ, Komuro H, Trapp BD (2010) Demyelination increases axonal stationary mitochondrial size and the speed of axonal mitochondrial transport. J Neurosci 30 (19):6658-6666. doi:10.1523/JNEUROSCI.5265-09.2010

8. Loew LM, Tuft RA, Carrington W, Fay FS (1993) Imaging in five dimensions: time-dependent membrane potentials in individual mitochondria. Biophys J 65 (6):2396-2407. doi:10.1016/S0006-3495(93)81318-3

9. Miller KE, Sheetz MP (2004) Axonal mitochondrial transport and potential are correlated. J Cell Sci 117 (Pt 13):2791-2804. doi:10.1242/jcs.01130

10. Ohno N, Kidd GJ, Mahad D, Kiryu-Seo S, Avishai A, Komuro H, Trapp BD (2011) Myelination and axonal electrical activity modulate the distribution and motility of mitochondria at CNS nodes of Ranvier. J Neurosci 31 (20):7249-7258. doi:10.1523/JNEUROSCI.0095-11.2011

11. Thompson RJ, Doran JF, Jackson P, Dhillon AP, Rode J (1983) PGP 9.5--a new marker for vertebrate neurons and neuroendocrine cells. Brain Res 278 (1-2):224-228. doi:10.1016/0006-8993(83)90241-X

12. Tian L, Hires SA, Mao T, Huber D, Chiappe ME, Chalasani SH, Petreanu L, Akerboom J, McKinney SA, Schreiter ER, Bargmann CI, Jayaraman V, Svoboda K, Looger LL (2009) Imaging neural activity in worms, flies and mice with improved GCaMP calcium indicators. Nat Methods 6 (12):875-881. doi:10.1038/nmeth.1398

13. Tschachler E, Reinisch CM, Mayer C, Paiha K, Lassmann H, Weninger W (2004) Sheet preparations expose the dermal nerve plexus of human skin and render the dermal nerve end organ accessible to extensive analysis. J Invest Dermatol 122 (1):177-182. doi:10.1046/j.0022-202X.2003.22102.x

14. Verburg J, Hollenbeck PJ (2008) Mitochondrial membrane potential in axons increases with local nerve growth factor or semaphorin signaling. J Neurosci 28 (33):8306-8315. doi:10.1523/JNEUROSCI.2614-08.2008

15. Wang X, Su B, Siedlak SL, Moreira PI, Fujioka H, Wang Y, Casadesus G, Zhu X (2008) Amyloid-beta overproduction causes abnormal mitochondrial dynamics via differential modulation of mitochondrial fission/fusion proteins. Proc Natl Acad Sci U S A 105 (49):19318-19323. doi:10.1073/pnas.0804871105





**Figure S1** The ratio of axonal to mitochondrial diameter is close to 3 in embryonic rat dorsal root ganglion cultures (DRGs). Embryonic (E16-17) rat DRGs were isolated, dissociated and cultured. Cultures were maintained as described in the above Methods for 1 month and were then subjected to embedding and sectioning for electron microscopy. **a** Ultrastructure of a demonstrative 1-month-old axon containing a mitochondrion (arrow). **b** The ratio between the diameters of axons and mitochondria is close to 3. The number of axons and mitochondria are shown. Scale bar: **a** 500nm

Submitted to Acta Neuropathologica

**Title: Mitochondrial Fission Augments Capsaicin-induced Axonal Degeneration**

**Authors:** Hao Chiang^1,2^, Nobuhiko Ohno^2^, Yu-Lin Hsieh^1^, Don J Mahad^2^, Shin Kikuchi^2^, Hitoshi Komuro^2^, Sung-Tsang Hsieh^1,3^, and Bruce D. Trapp^2^

**Affiliations:** ^1^Department of Anatomy and Cell Biology, National Taiwan University College of Medicine, Taipei, 10051, Taiwan; ^2^Department of Neurosciences, Lerner Research Institute, Cleveland Clinic, Cleveland, OH 44195**;** ^3^Department of Neurology, National Taiwan University Hospital, Taipei, 10002, Taiwan

**Corresponding authors:** Dr. Bruce D. Trapp ([trappb@ccf.org](mailto:trappb@ccf.org)); Dr. Sung-Tsang Hsieh ([shsieh@ntu.edu.tw](mailto:shsieh@ntu.edu.tw))

**
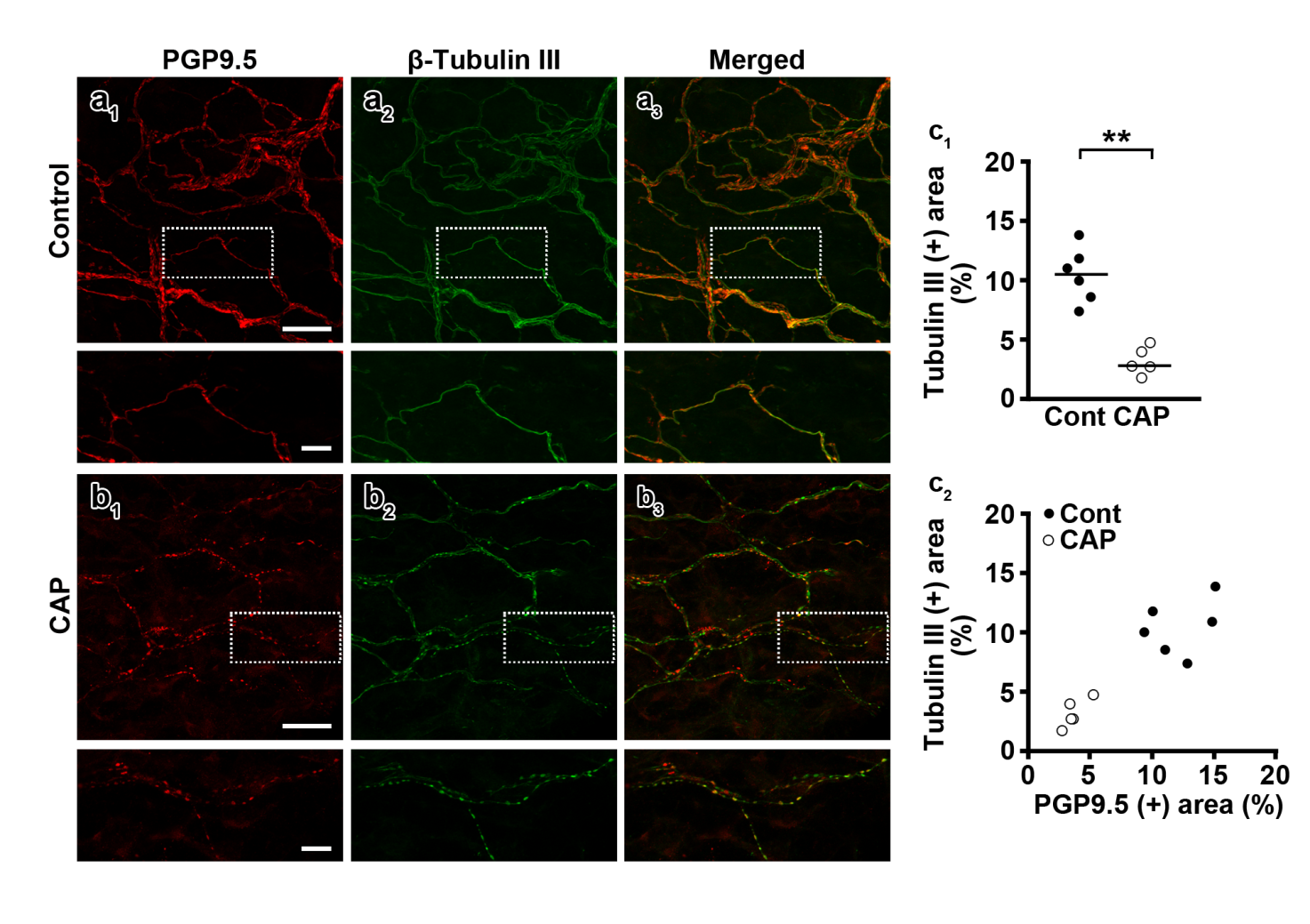
**

**Online Resource 2** Capsaicin induces axonal loss in the dermis. Dermal axons were examined with protein gene product 9.5 (PGP9.5, red) and an axonal structural marker, ß-tubulin isotype III (ß-tubulin III, green). **a-b** The expression of PGP9.5 was highly co-localized with ß-tubulin III in both control nerves (**a**_1_-**a_3_**) and capsaicin-treated nerves (**b_1_**-**b_3_**). The boxed areas are magnified in the corresponding lower panels. Capsaicin induced axonal beading (**b_1_-b_3_**, insets) and axonal loss (**c_1_**) (**, P < 0.01 by Mann-Whitney test). **c_2_** PGP9.5-positive area was highly correlated with ß-tubulin III-positive area. Dots represent individual subjects. (Pearson γ = 0.903, p = 0.0001). Scale bars: **a_1_-b_3_** upper panels 25μm; lower panels 10μm

Submitted to Acta Neuropathologica

**Title: Mitochondrial Fission Augments Capsaicin-induced Axonal Degeneration**

**Authors:** Hao Chiang^1,2^, Nobuhiko Ohno^2^, Yu-Lin Hsieh^1^, Don J Mahad^2^, Shin Kikuchi^2^, Hitoshi Komuro^2^, Sung-Tsang Hsieh^1,3^, and Bruce D. Trapp^2^

**Affiliations:** ^1^Department of Anatomy and Cell Biology, National Taiwan University College of Medicine, Taipei, 10051, Taiwan; ^2^Department of Neurosciences, Lerner Research Institute, Cleveland Clinic, Cleveland, OH 44195**;** ^3^Department of Neurology, National Taiwan University Hospital, Taipei, 10002, Taiwan

**Corresponding authors:** Dr. Bruce D. Trapp ([trappb@ccf.org](mailto:trappb@ccf.org)); Dr. Sung-Tsang Hsieh ([shsieh@ntu.edu.tw](mailto:shsieh@ntu.edu.tw))





**Online Resource 3** Capsaicin induces axonal degeneration. **a-b** Stitches of high magnification electron microscopic images in Fig.1**c_1_** and **c_2_** are shown in **a** and **b**, respectively, to demonstrate unmyelinated axonal degeneration and mitochondrial damage in capsaicin-treated medial plantar nerves (**b**) compared to control nerves (**a**). Scale bars: **a-b** 200 nm

**Title: Mitochondrial Fission Augments Capsaicin-induced Axonal Degeneration**

**Authors:** Hao Chiang^1,2^, Nobuhiko Ohno^2^, Yu-Lin Hsieh^1^, Don J Mahad^2^, Shin Kikuchi^2^, Hitoshi Komuro^2^, Sung-Tsang Hsieh^1,3^, and Bruce D. Trapp^2^

**Affiliations:** ^1^Department of Anatomy and Cell Biology, National Taiwan University College of Medicine, Taipei, 10051, Taiwan; ^2^Department of Neurosciences, Lerner Research Institute, Cleveland Clinic, Cleveland, OH 44195**;** ^3^Department of Neurology, National Taiwan University Hospital, Taipei, 10002, Taiwan

**Corresponding authors:** Dr. Bruce D. Trapp ([trappb@ccf.org](mailto:trappb@ccf.org)); Dr. Sung-Tsang Hsieh ([shsieh@ntu.edu.tw](mailto:shsieh@ntu.edu.tw))


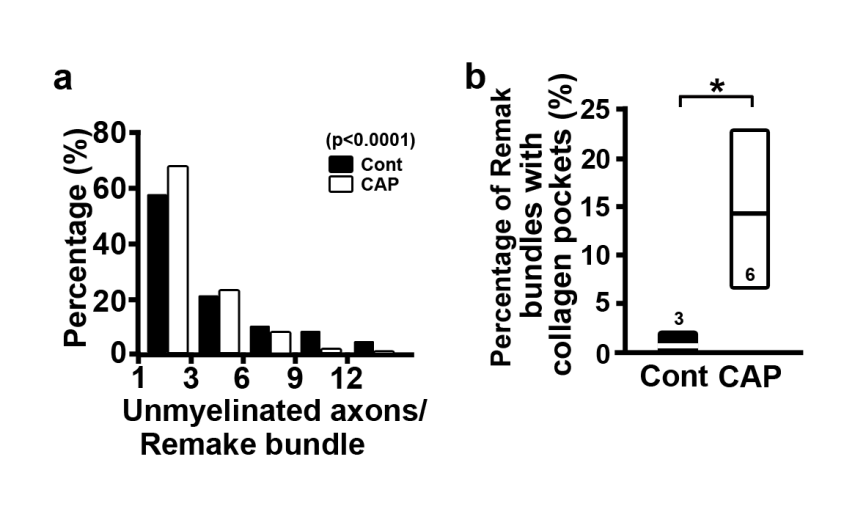


**Online Resource 4** Capsaicin induces unmyelinated axonal degeneration in medial plantar nerves. **a** Number of intact unmyelinated axons in Remak bundles were counted and sub-grouped. Histograms show reduced axons in Remak bundles after capsaicin treatment. (*χ*² = 32, P < 0.0001; Chi-squared test). Remak bundles from 3 control (Cont) and 6 capsaicin (CAP) subjects were pooled. **b** The percentage of Remak bundles containing empty collagen pockets was increased after capsaicin treatment. (*, P = 0.0126; unpaired *t*-test).

Submitted to Acta Neuropathologica

**Title: Mitochondrial Fission Augments Capsaicin-induced Axonal Degeneration**

**Authors:** Hao Chiang^1,2^, Nobuhiko Ohno^2^, Yu-Lin Hsieh^1^, Don J Mahad^2^, Shin Kikuchi^2^, Hitoshi Komuro^2^, Sung-Tsang Hsieh^1,3^, and Bruce D. Trapp^2^

**Affiliations:** ^1^Department of Anatomy and Cell Biology, National Taiwan University College of Medicine, Taipei, 10051, Taiwan; ^2^Department of Neurosciences, Lerner Research Institute, Cleveland Clinic, Cleveland, OH 44195**;** ^3^Department of Neurology, National Taiwan University Hospital, Taipei, 10002, Taiwan

**Corresponding authors:** Dr. Bruce D. Trapp ([trappb@ccf.org](mailto:trappb@ccf.org)); Dr. Sung-Tsang Hsieh ([shsieh@ntu.edu.tw](mailto:shsieh@ntu.edu.tw))


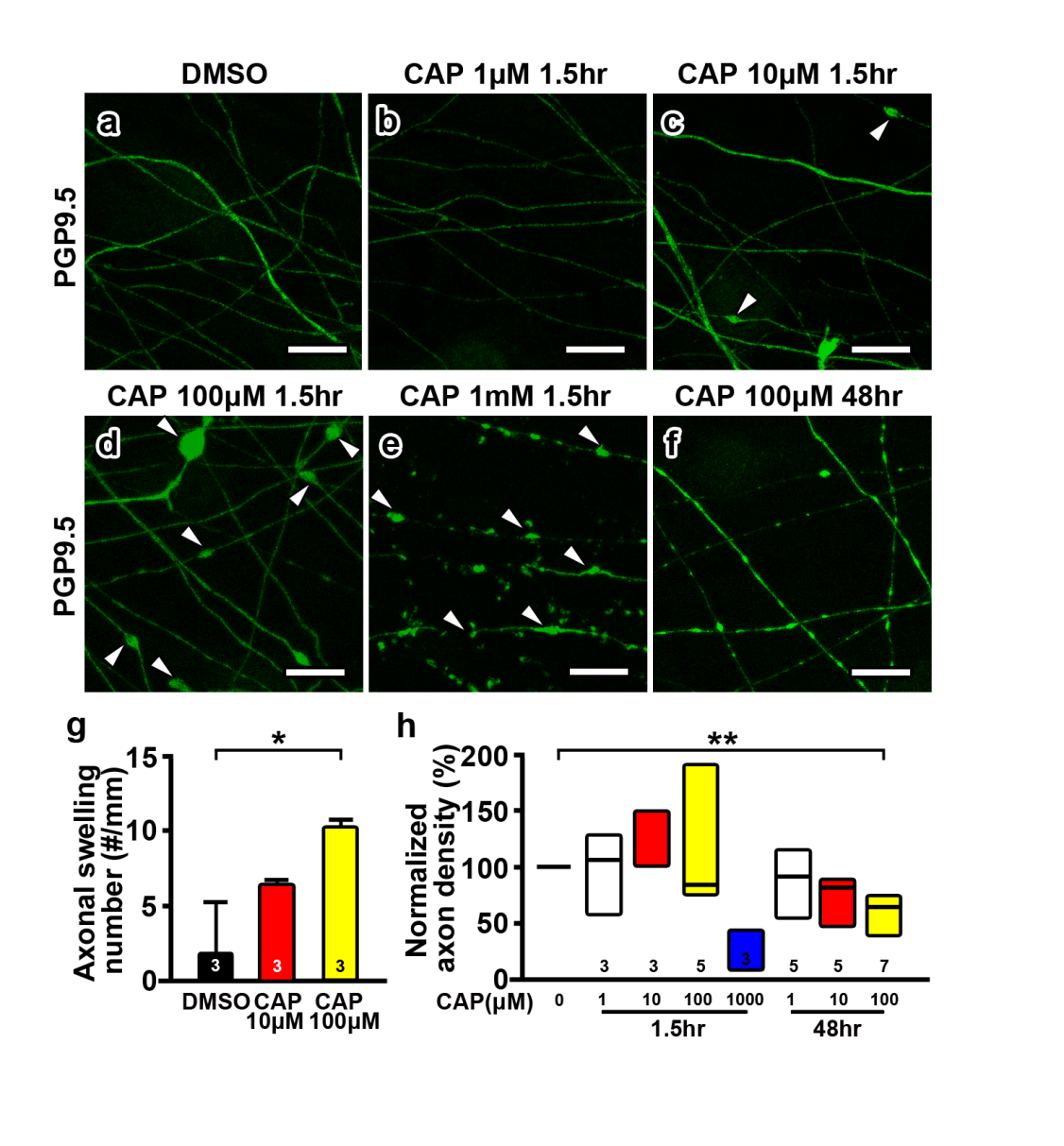


**Online Resource 5** Capsaicin induces axonal ovoid formation in a dose-dependent manner. **a-f** Confocal images of dorsal root ganglia (DRG) axons labeled with PGP9.5 demonstrate that axonal ovoid number is dose-dependent on capsaicin (CAP) concentration and that axonal density is reduced with longer incubation times. **g** 100 μM capsaicin significantly induced axonal ovoid formation compared to the effects of DMSO (*, P < 0.05 by Kruskal-Wallis test and Dunn’s multiple comparison test). **h** Histograms show that axonal density, which is normalized to that of DMSO, decreases 48 hours after capsaicin treatment (**, P < 0.01 by Kruskal-Wallis test and Dunn’s multiple comparison test). Analysis was based on 3-4 randomly chosen fields in each replicate. The numbers of independent experiments are shown in the bar graphs. Scale bars: **a-f** 10 μm

Submitted to Acta Neuropathologica

**Title: Mitochondrial Fission Augments Capsaicin-induced Axonal Degeneration**

**Authors:** Hao Chiang^1,2^, Nobuhiko Ohno^2^, Yu-Lin Hsieh^1^, Don J Mahad^2^, Shin Kikuchi^2^, Hitoshi Komuro^2^, Sung-Tsang Hsieh^1,3^, and Bruce D. Trapp^2^

**Affiliations:** ^1^Department of Anatomy and Cell Biology, National Taiwan University College of Medicine, Taipei, 10051, Taiwan; ^2^Department of Neurosciences, Lerner Research Institute, Cleveland Clinic, Cleveland, OH 44195**;** ^3^Department of Neurology, National Taiwan University Hospital, Taipei, 10002, Taiwan

**Corresponding authors:** Dr. Bruce D. Trapp ([trappb@ccf.org](mailto:trappb@ccf.org)); Dr. Sung-Tsang Hsieh ([shsieh@ntu.edu.tw](mailto:shsieh@ntu.edu.tw))


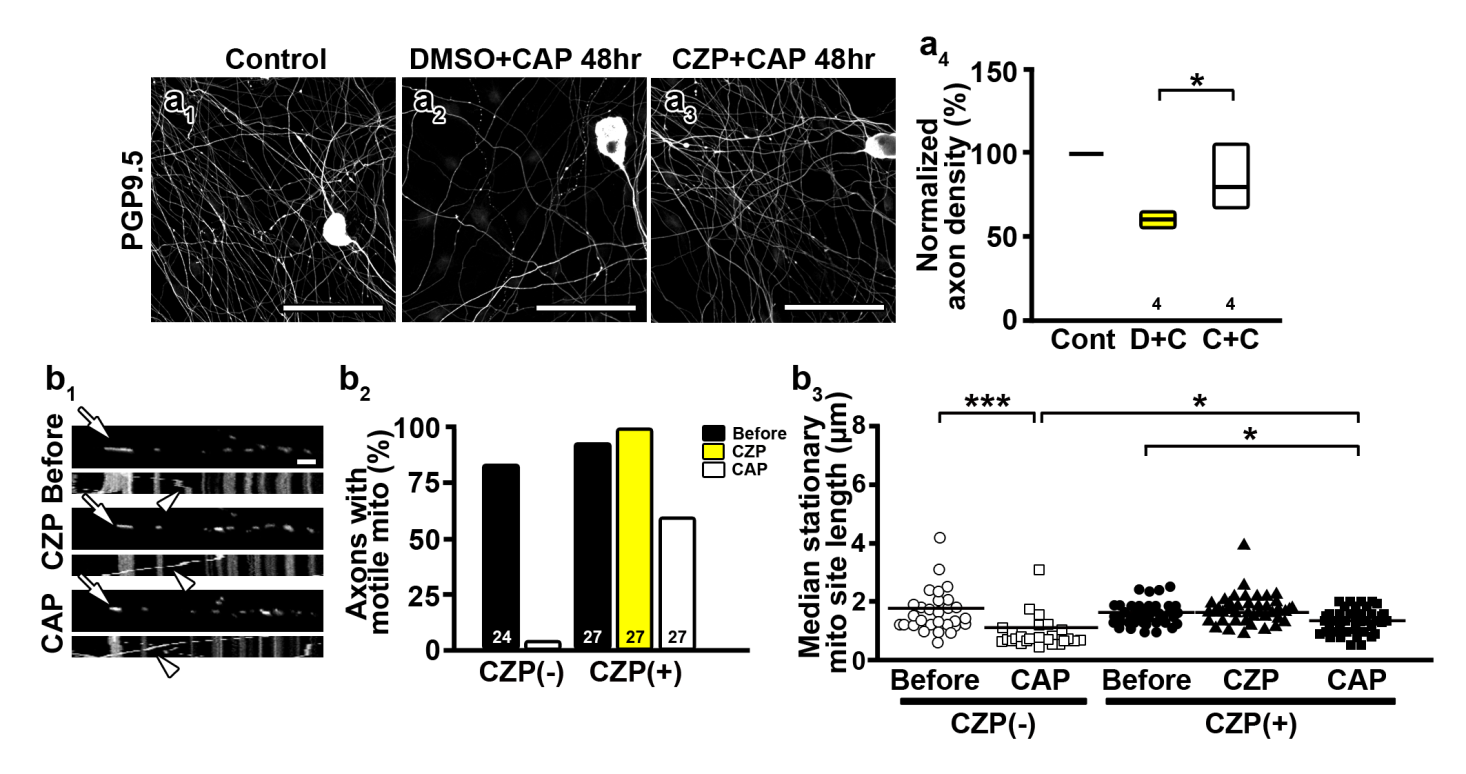


**Online Resource 6** The capsaicin antagonist capsazepine (CZP) rescues the reduced motility of axonal mitochondria and the axonal loss caused by capsaicin treatment. **a_1_-a_4_** CZP pretreatment rescued axonal loss 48 hours after capsaicin treatment. Cont = Control; D + C = DMSO + CAP; C + C = CZP + CAP (*, P < 0.05 by Mann-Whitney test). **b_1_** Representative frames of time-lapse images of axonal mitochondrial transport and resulting kymographs before CZP, 10 minutes during CZP administration, and 30 minutes after capsaicin treatment. The length (arrows) and motility (arrowheads) of mitochondria were maintained when CZP was added prior to capsaicin treatment. **b_2_** Pretreatment with CZP [(CZP(+)] rescued the reduced motility of axonal mitochondria as compared to control studies without CZP pretreatment [(CZP(-)] as demonstrated in Fig. 4. **b_3_** Pretreatment of CZP rescued mitochondrial fragmentation upon capsaicin treatment (*, P < 0.05; ***, P < 0.0001 by Kruskal-Wallis test and Dunn’s multiple comparison test). The numbers of independent experiments and axons are shown in the bar graphs in **a_4_** and **b_2_**, respectively. Scale bars: **a_1_-a_3_** 100 μm; **b_1_** 5 μm

Submitted to Acta Neuropathologica

**Title: Mitochondrial Fission Augments Capsaicin-induced Axonal Degeneration**

**Authors:** Hao Chiang^1,2^, Nobuhiko Ohno^2^, Yu-Lin Hsieh^1^, Don J Mahad^2^, Shin Kikuchi^2^, Hitoshi Komuro^2^, Sung-Tsang Hsieh^1,3^, and Bruce D. Trapp^2^

**Affiliations:** ^1^Department of Anatomy and Cell Biology, National Taiwan University College of Medicine, Taipei, 10051, Taiwan; ^2^Department of Neurosciences, Lerner Research Institute, Cleveland Clinic, Cleveland, OH 44195**;** ^3^Department of Neurology, National Taiwan University Hospital, Taipei, 10002, Taiwan

**Corresponding authors:** Dr. Bruce D. Trapp ([trappb@ccf.org](mailto:trappb@ccf.org)); Dr. Sung-Tsang Hsieh ([shsieh@ntu.edu.tw](mailto:shsieh@ntu.edu.tw))


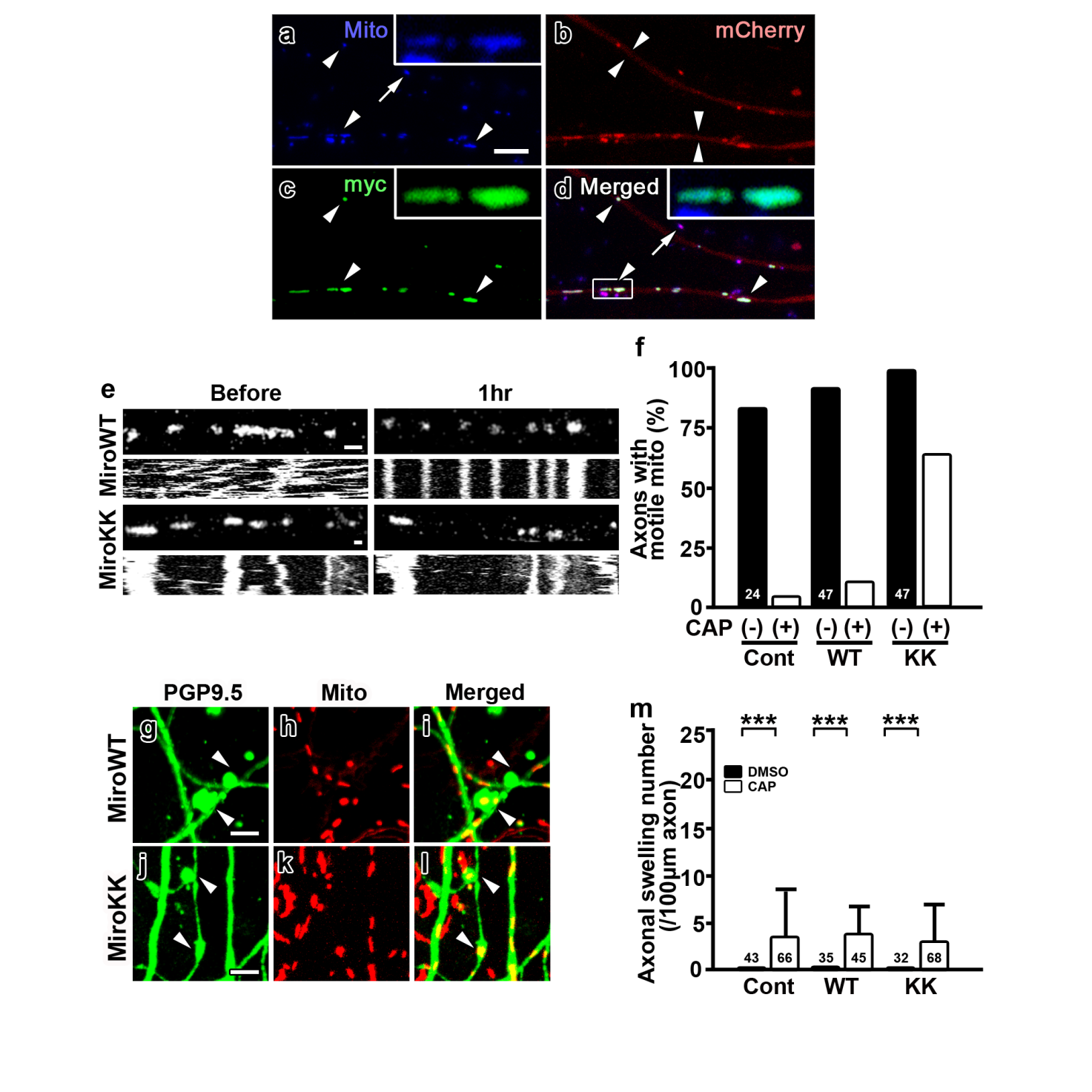


**Online Resource 7** Sustained mitochondrial motility upon capsaicin treatment does not affect axonal ovoid formation. **a-d** Confocal images show the expression of wild-type Miro (MiroWT) or EF-hand mutant (MiroKK) in dorsal root ganglia (DRG) axons. Miro transfection in axons is demonstrated by the co-localization of mitochondria (**a**, arrowheads), *myc*-tag protein labeling (**c**, arrowheads), and axoplasmic mCherry expression (**b**, arrowheads). Mitochondria labeled with mCherry but not *myc*-tag (**a**, **b**, arrow) are not regarded as axonal mitochondria. **e-f** Representative frames of time-lapse imaging and resulting kymographs of axonal mitochondria transfected with MiroWT (WT) or MiroKK (KK) before (-) and after capsaicin treatment (CAP) (1 hour in **e**­­­, and (+) in **f**). Motility of mitochondria in control and MiroWT-overexpressed axons, but not in MiroKK-transfected axons, was decreased after capsaicin treatment. **g-l** Confocal images of Mito-Dendra2 labeling (Mito) and PGP9.5 immunostaining after capsaicin treatment. Axonal swellings were increased in both MiroWT- and MiroKK-transfected axons after CAP (**g-l**, arrowheads). **m** There was no significant difference in the number of axonal swellings among the three groups (***, P < 0.0001 by Kruskal-Wallis test and Dunn’s multiple comparison test). The numbers of axons analyzed are shown in the corresponding bar graphs. Scale bars: **a** 5 μm; **e** 1 μm; **g-l** 4 μm

Submitted to Acta Neuropathologica

**Title: Mitochondrial Fission Augments Capsaicin-induced Axonal Degeneration**

**Authors:** Hao Chiang^1,2^, Nobuhiko Ohno^2^, Yu-Lin Hsieh^1^, Don J Mahad^2^, Shin Kikuchi^2^, Hitoshi Komuro^2^, Sung-Tsang Hsieh^1,3^, and Bruce D. Trapp^2^

**Affiliations:** ^1^Department of Anatomy and Cell Biology, National Taiwan University College of Medicine, Taipei, 10051, Taiwan; ^2^Department of Neurosciences, Lerner Research Institute, Cleveland Clinic, Cleveland, OH 44195**;** ^3^Department of Neurology, National Taiwan University Hospital, Taipei, 10002, Taiwan

**Corresponding authors:** Dr. Bruce D. Trapp ([trappb@ccf.org](mailto:trappb@ccf.org)); Dr. Sung-Tsang Hsieh ([shsieh@ntu.edu.tw](mailto:shsieh@ntu.edu.tw))


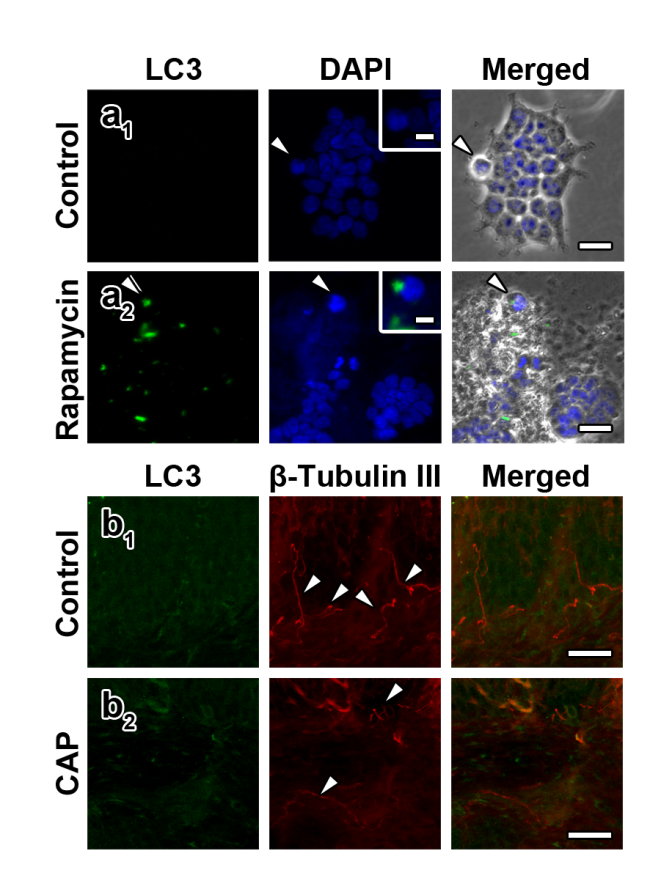


**Online Resource 8** Autophagy is not detected in capsaicin-treated dermal nerves. Autophagy was evaluated with Light Chain 3 (LC3), an indicator for autophagosomes. **a_1_-a_2_** Autophagy was induced in 293T cells with 1μM rapamycin treatment for 6 h as a positive control. In a representative 293T cell (**a_2_**, insets) labeled with DAPI (arrowheads), LC3-positive vesicles (double arrowheads) were only evident after rapamycin treatment compared to control (**a_1_**, insets). **b_1_-b_2_** There were no LC3-positive vesicles in control or capsaicin-treated dermal nerves identified with ß-tubulin isotype III antibodies (ß-tubulin III, arrowheads). Scale bars: **a_1_-a_2_** 25 μm; **insets** 10μm; **b_1_-b_2_** 25 μm
